# Anaplastic transformation in thyroid cancer revealed by single-cell transcriptomics

**DOI:** 10.1172/JCI169653

**Published:** 2023-06-01

**Authors:** Lina Lu, Jennifer Rui Wang, Ying C. Henderson, Shanshan Bai, Jie Yang, Min Hu, Cheng-Kai Shiau, Timothy Pan, Yuanqing Yan, Tuan M. Tran, Jianzhuo Li, Rachel Kieser, Xiao Zhao, Jiping Wang, Roza Nurieva, Michelle D. Williams, Maria E. Cabanillas, Ramona Dadu, Naifa Lamki Busaidy, Mark Zafereo, Nicholas Navin, Stephen Y. Lai, Ruli Gao

**Affiliations:** 1 Department of Biochemistry and Molecular Genetics, and; 2 Center for Cancer Genomics, Robert H. Lurie Cancer Center, Northwestern University, Chicago, Illinois, USA.; 3 Department of Head and Neck Surgery,; 4 Department of Genetics, and; 5 Department of Genitourinary Medical Oncology, University of Texas (UT) MD Anderson Cancer Center, Houston, Texas, USA.; 6 Department of Radiation Oncology, New York University Langone School of Medicine, New York, New York, USA.; 7 The Driskill Graduate Program and; 8 Department of Surgery, Northwestern University, Chicago, Illinois, USA.; 9 Center for RNA Therapeutics, Department of Cardiovascular Sciences, and; 10 Center for Bioinformatics and Computational Biology, Department of Cardiovascular Sciences, Houston Methodist Research Institute, Houston, Texas, USA.; 11 Department of Immunology,; 12 Department of Pathology, and; 13 Department of Endocrine Neoplasia and Hormonal Disorders, UT MD Anderson Cancer Center, Houston, Texas, USA.; 14 Graduate School of Biological Sciences, University of Texas, Houston, Texas, USA.; 15 Department of Bioinformatics and Computational Biology,; 16 Department of Radiation Oncology, and; 17 Department of Molecular and Cellular Oncology, UT MD Anderson Cancer Center, Houston, Texas, USA.

**Keywords:** Genetics, Oncology, Bioinformatics, Expression profiling, Head and neck cancer

## Abstract

The deadliest anaplastic thyroid cancer (ATC) often transforms from indolent differentiated thyroid cancer (DTC); however, the complex intratumor transformation process is poorly understood. We investigated an anaplastic transformation model by dissecting both cell lineage and cell fate transitions using single-cell transcriptomic and genetic alteration data from patients with different subtypes of thyroid cancer. The resulting spectrum of ATC transformation included stress-responsive DTC cells, inflammatory ATC cells (iATCs), and mitotic-defective ATC cells and extended all the way to mesenchymal ATC cells (mATCs). Furthermore, our analysis identified 2 important milestones: (a) a diploid stage, in which iATC cells were diploids with inflammatory phenotypes and (b) an aneuploid stage, in which mATCs gained aneuploid genomes and mesenchymal phenotypes, producing excessive amounts of collagen and collagen-interacting receptors. In parallel, cancer-associated fibroblasts showed strong interactions among mesenchymal cell types, macrophages shifted from M1 to M2 states, and T cells reprogrammed from cytotoxic to exhausted states, highlighting new therapeutic opportunities for the treatment of ATC.

## Graphical abstract

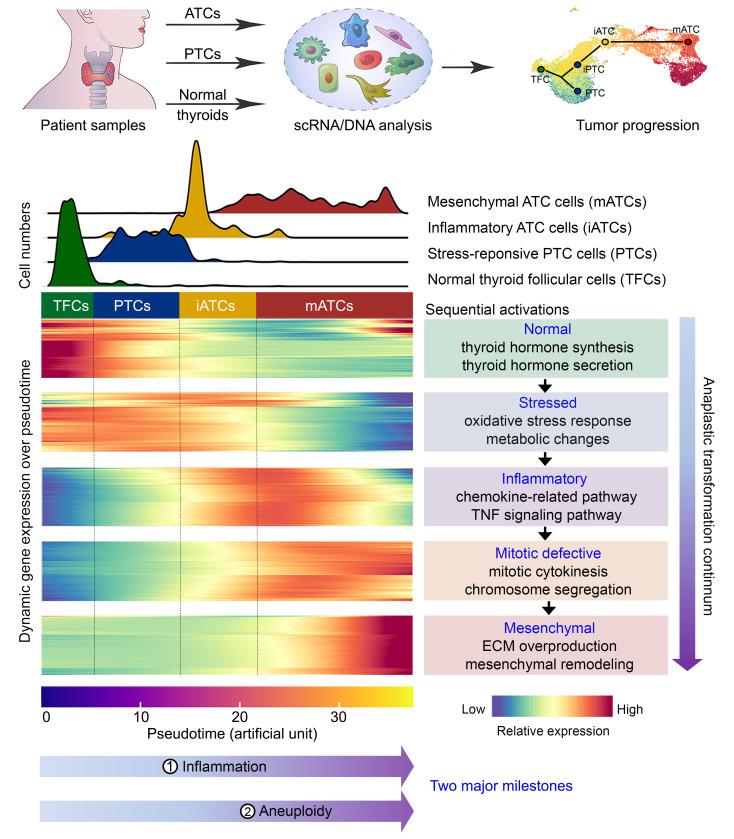

## Introduction

Anaplastic thyroid cancer (ATC), characterized by an undifferentiated morphology, is arguably one of the most lethal malignancies. According to Surveillance, Epidemiology and End Results (SEER) data (released April 2021) (1), the 5-year survival rate for ATC patients with all SEER stages combined is 7%, accounting for approximately 40% of all thyroid cancer–related mortalities. Treatment options for ATC have been limited (2–5) until recent FDA approval of dabrafenib and trametinib for the treatment of patients with *BRAF V600E*–mutated ATC (6, 7). Unfortunately, for the non-*BRAF*-mutated patient population, no systematic treatment is available. In contrast, differentiated thyroid cancers (DTCs), most commonly (~90%) papillary thyroid carcinoma (PTC), often exhibit indolent behavior (99.7% combined survival rate >5 years) (8–11). Approximately 20% of patients with ATC report a prior history of DTC, and up to 70% of patients are found to have synchronous DTC regions on histopathology (12–14), suggesting an intratumor evolution process from DTC to ATC. Identification of the driving forces of anaplastic transformation from DTC to ATC is urgently needed to discover new effective therapies for ATC. However, for decades, the rarity of ATC and the short survival time impeded progress in understanding this highly lethal transformation process.

Previous bulk genome-sequencing studies have detected extensive genetic alterations in ATC, including *BRAF*, *RAS*, *TP53*, and *TERT* mutations and others in the PI3K/AKT pathway (15–21). Additionally, ATC tumors often have aneuploidy with extensive large-scale copy number alterations (CNAs) (21, 22). In contrast, the overall somatic mutation burden in DTC is significantly less, except in the case of frequent *BRAF* mutations (15–21, 23). Moreover, the transcriptional spectrum along the evolutionary progression of ATC remains unclear.

In this study, we combined single-cell transcriptomes, bulk transcriptomes, and targeted mutation data from both in-house and publicly available data sets to construct an anaplastic transformation continuum that starts from normal thyrocytes and extends all the way to the ATC tumor cells that conferred the high lethality. We defined key milestones of tumor cells as well as the cellular network in the tumor microenvironment (TME) during anaplastic transformation, providing evidence for the development of new therapeutics for patients with ATC.

## Results

### An overview of evolving single-cell ecosystems during thyroid cancer progression.

**Figure 1:**
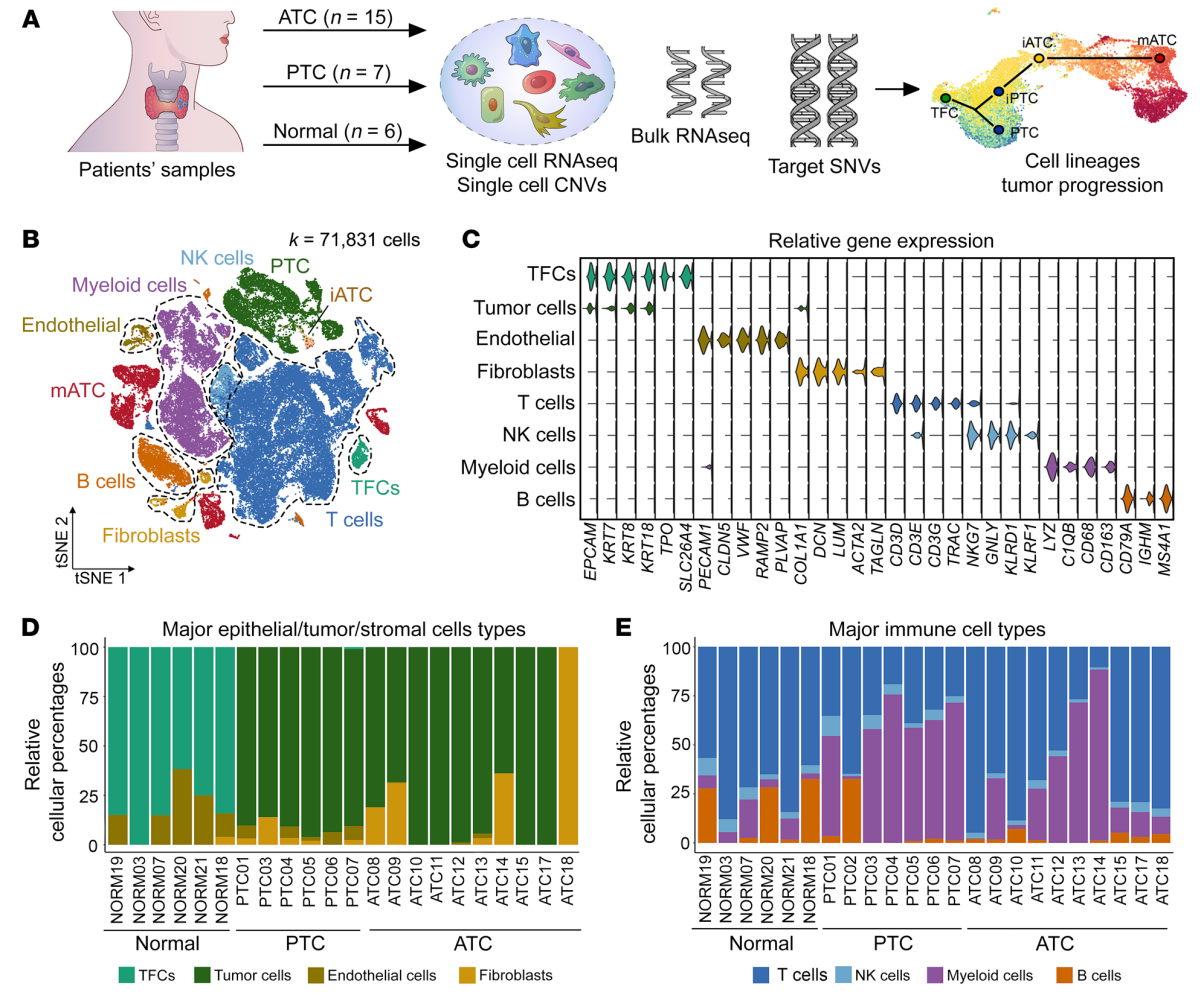
Single-cell profiles of thyroid tumor and normal tissues. (**A**) Study design for all in-house data. (**B**) *t*SNE projection of single cells annotated with major cell types. Tumor cell subtypes (PTC, iATC, mATC) are color coded. (**C**) Violin plots of marker gene expression in 8 major cell types. (**D** and **E**) Stack bar plot of relative frequencies of nonimmune cells (**D**) and immune cells (**E**) in individual samples.

To investigate the dynamic changes within the cellular ecosystem during thyroid cancer progression, we performed high-throughput 3′single-cell RNA-Seq (scRNA-Seq) (10X Genomics) for PTC and ATC tumors as well as adjacent normal thyroid tissue (Figure 1A and Supplemental Table 1; supplemental material available online with this article; https://doi.org/10.1172/JCI169653DS1). Morphologically, the PTC samples in this cohort included 5 conventional, 1 follicular, and 1 tall cell subtype based on their histological characteristics (Supplemental Figure 1, A–C). Among the ATC samples, 3 showed nested epithelial clusters with squamoid features and mixed inflammation (Supplemental Figure 2A), and 6 showed sheet-like growth of tumor cells with a spindled morphology (Supplemental Figure 2B). After sequencing, we first identified the major cell types within individual patients to avoid clustering artifacts caused by data integration, followed by identification of subpopulations through reclustering of same-cell types. We combined the automated prediction using singleR (24) with manual curation using known markers to define 8 major cell types (Figure 1, B and C), including normal thyroid follicular cells (TFCs), tumor cells, endothelial cells, fibroblasts, T cells, NK cells, myeloid cells, and B cells. Both TFCs and tumor cells expressed epithelial markers due to shared epithelial origins. To mitigate this, we classified normal TFCs using 3 additional markers (i.e., *TFF3*, *TPO*, *SLC26A4*) that were previously shown to be expressed in normal thyroids (25) but not in tumor cells.

We compared the relative frequencies of different cell types in ATC, PTC, and normal thyroids to mitigate sampling biases across patients (see Supplemental Table 2 for the absolute number of cells for each cell type). Our data showed that tumor cells accounted for most nonimmune cell components in both ATC and PTC tumors (Figure 1D). Interestingly, both ATC and PTC tumors had significantly fewer endothelial cells compared with normal thyroids (2-sided *t* test *P* = 0.058 and 0.023, respectively). Among all immune cell types, we observed significant increases in myeloid cells in both ATC and PTC (2-sided *t* test *P =* 0.05 and 0.002, respectively) and a decrease in T cells in PTC (2-sided *t* test *P =* 0.0006) but not ATC compared with normal tissues (Figure 1E), implying the important roles of macrophages and T cells in thyroid cancer.

### Machine learning and characterization of tumor cell subtypes in thyroid cancer.

**Figure 2:**
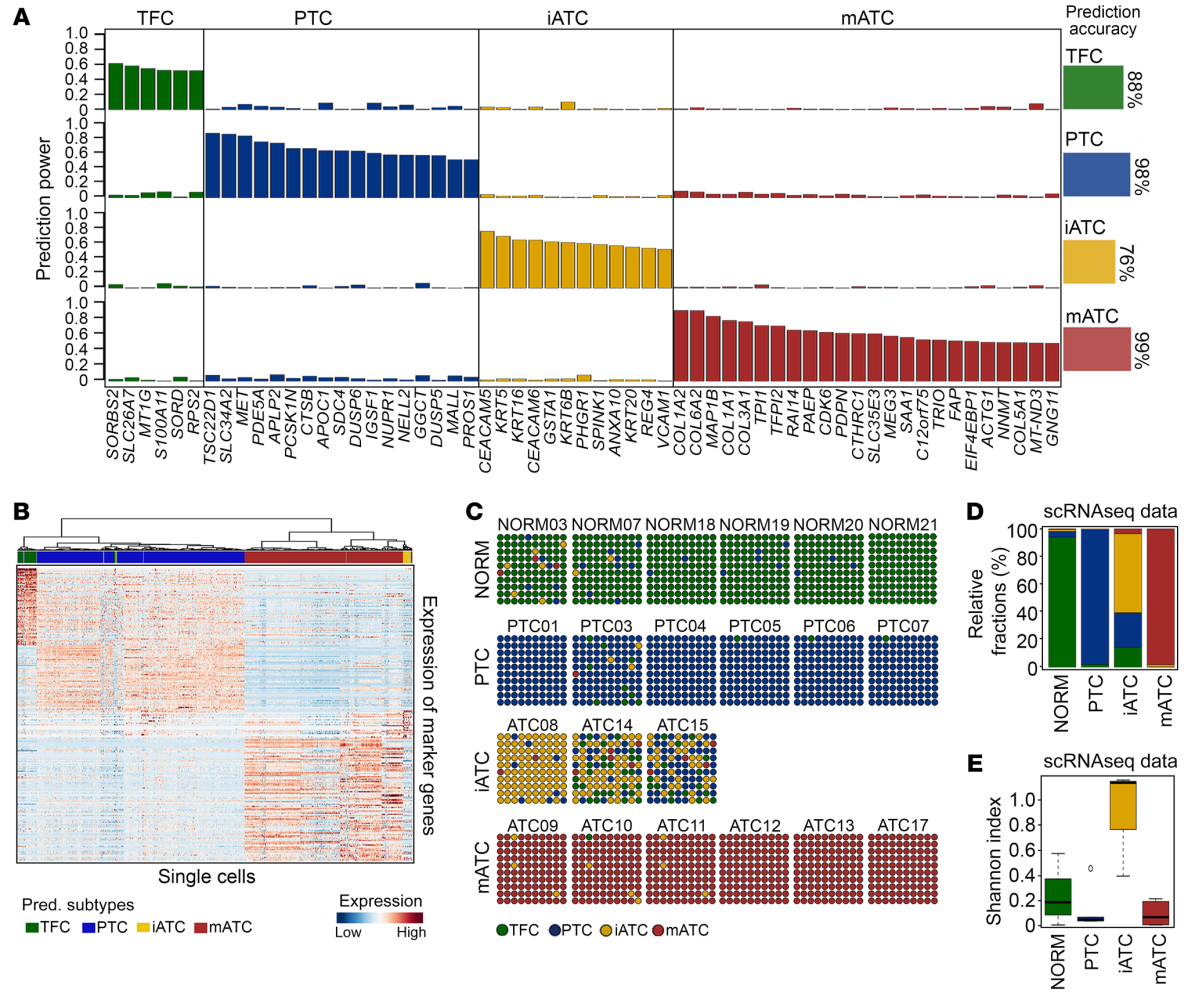
Coexistence of epithelial cell subtypes in thyroid cancer. (**A**) Bar plot of the prediction powers of a 59-gene predictor selected by the lasso model, with the averaged prediction accuracies per subtype. (**B**) Heatmap showing unsupervised clustering of single cells. (**C**) Dot plots depicting predicted subtype compositions in each sample. Each dot represents 1% of the cells. (**D**) Stacked bar plot of relative fractions of 4 epithelial cell subtypes. (**E**) Box plot of the Shannon Diversity Index. All boxes are centered at the median and bounded by the first (Q1) and third (Q3) quartiles. Upper whiskers indicate the minimum (maximum, Q3 + 1.5 IQR), and lower whiskers indicate the maximum (minimum, Q1 – 1.5 IQR). NORM, normal.

We built an integrative machine-learning and clustering tool, scTypeTC, to classify the molecular subtypes of tumor cells using single-cell transcriptomic data. To mitigate gene dropout issues in single cells, we first grouped all TFC and tumor cells from individual patients into major transcriptional clusters, where most patients had only 1 cluster except for 1 ATC sample and 3 PTC samples that had 2 transcriptional clusters, named c1 and c2. We then calculated the consensus transcriptional profiles of these individual clusters and reclustered them into 4 major clusters (Supplemental Figure 4, A–C, and Supplemental Table 3). These included a cluster of mesenchymal ATC cells (mATCs) that overexpressed mesenchymal genes (e.g., *ZEB2*, *MMPs*, *TGFB1*, *VCAN*, *COL6A3*), a cluster of inflammatory ATC cells (iATCs) that overexpressed inflammatory genes (e.g., *S100A9/10*, *IFI27*, *AGR2*, *CEACAM1/5/6*, *AKR1B1/10*), a cluster of classical PTC cells that overexpressed stress-responsive and metabolic/catabolic genes that were previously reported (23), and a cluster of normal TFCs. Next, we applied the glmnet (26) lasso model to select variable genes to predict subtypes (Figure 2A), which resulted in a list of 59 genes that had greater than 50% specificity and less than 10% nonspecificity in predicting subtypes of more than 1,000 iterations of gradient regularization. Last, we loaded the 59 gene predictor onto an optimal lasso model to predict single-cell subtypes. Cells with less than 50% consistent predictions were labeled as undefined. Comparison of the scTypeTC prediction with manually annotated clustering results (Figure 2, A and B, and Supplemental Figure 4D) revealed a high concordance (98% on average). We noted that iATC cells had a lower prediction accuracy (76%) than other subtypes, largely due to the smaller numbers of iATC cells, gene dropouts, and relatively fewer number of genes in the signature.

Our subtyping results showed that thyroid tumors had a wide range of intratumoral heterogeneity in epithelial components (Figure 2, C–E). In this cohort, 6 ATC tumors were predicted as being of the mATC subtype, which was dominated by mATC cells intermixed with small fractions of iATC cells, with 3 tumors (ATC12, ATC13, ATC17) being purely mATC. In contrast, the iATC tumors harbored mixtures of 3 subtypes of tumor cells and had the highest intradiversity score (*t* test *P* < 0.001, Figure 2E), which provided a great opportunity to infer tumor progression. Our prediction results are consistent with immunohistopathologic morphologies, which showed that 3 iATC tumors had inflammation and nested growth patterns (Supplemental Figure 2A), whereas 6 mATC tumors had mesenchymal phenotypes and a uniform tumor cell appearance (Supplemental Figure 2B). The PTC tumors were relatively more homogeneous, with the exception of PTC03, which harbored rare ATC cells (~4%). Interestingly, this tumor had tall cell morphology (Supplemental Figure 1C) that is considered a more aggressive subtype, which was confirmed by our clinical data (invasive and fast growth).

**Figure 3:**
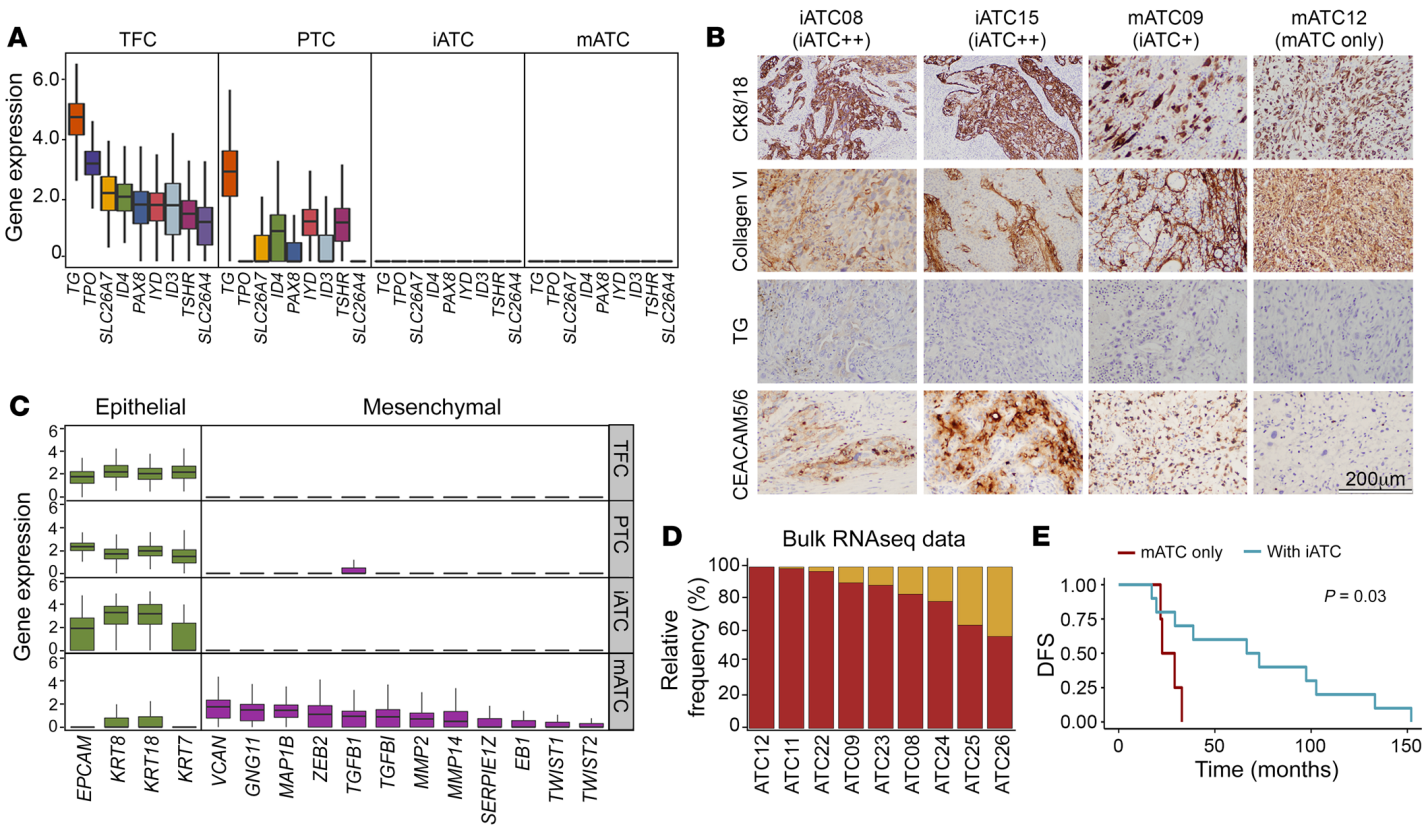
Characteristics of epithelial cell subtypes in thyroid cancer. (**A**) Box plot of thyroid function gene expression. (**B**) IHC staining of protein markers in 4 ATC samples. Scale bar: 200 μm. (**C**) Box plots of epithelial (green) and mesenchymal (purple) marker gene expression. (**D**) Stacked bar plot of relative fractions of 2 ATC subtypes. (**E**) Kaplan-Meier plot of disease-free survival (DFS) of 14 patients with ATC. *P* values were determined by 2-sided log-rank test. All boxes are centered at the median and bounded by the first (Q1) and third (Q3) quartiles. Upper whiskers show the minimum (maximum, Q3 + 1.5 IQR), and lower whiskers show the maximum (minimum, Q1 – 1.5 IQR).

We further characterized the 2 ATC subtypes using both gene expression and IHC analyses (Figure 3, A–C, Supplemental Figure 4, E and F, and Supplemental Table 4). In keeping with the behavior of ATCs as undifferentiated tumors, both mATC and iATC cells lost expression of thyroid differentiation genes (Figure 3A). The IHC staining results validated that TG, a marker of thyroid differentiation, was lost in all tested ATC samples (Figure 3B), and the other 2 thyroid differentiation markers, PAX8 and TTF1, had weak to null intensities (Supplemental Figure 4E). Furthermore, gene expression analysis demonstrated that mATC cells overexpressed many mesenchymal genes such as mesenchymal transcription factor genes (*ZEB2*, *TWIST1*), extracellular matrix factor genes (*VCAN*, *GNG11*, *MAP1B*, *MMP2*), and epithelial-mesenchymal transition (EMT) regulator genes (*TGFB1*, *TGFBI*) (Figure 3C). Notably, mATC cells also overexpressed many collagen genes (Figure 2A and Supplemental Figure 4A) that may further promote EMT, extracellular matrix (ECM) remodeling, and cell-cell communication (Supplemental Figure 4F). To determine whether the detection of collagen genes in mATC cells was due to floating RNAs from low-quality cells, we subjected all data to ambient RNA analysis using SoupX (27), which identified 2 samples that were affected (Supplemental Figure 4G). Even so, mATC cells retained high levels of collagen and mesenchymal gene expression after data cleanup with SoupX (27). Furthermore, our IHC results confirmed the excessive production of collagens in the ATC samples (Figure 3B). In concert with the mesenchymal phenotypes, mATC cells showed weak expression of epithelial marker genes such as *EPCAM* and *KRT8* (Figure 3C), suggesting that these cells underwent EMT. Comparing with mATC cells, iATC cells expressed a strong epithelial phenotype (KRT8^+^/KRT18^+^) (Figure 3, B and C) and high inflammatory programs but not EMT pathways (Figure 2A and Supplemental Figure 4, C and F). We validated the inflammatory features of iATC cells using IHC staining with CEACAM5/6, which revealed strong nested staining in iATC tumors, weak defused staining in iATC-containing mATC tumors, and no staining in pure mATC tumors (Figure 3B), consistent with subtype prediction results.

To further investigate the prevalence of iATC cells, we performed bulk RNA-Seq on 9 patient-derived ATC cell lines, including 4 cell lines that had matched scRNA-Seq data to validate the concordance of our analysis. We decomposed the bulk RNA-Seq data to calculate the relative composition of the iATC and mATC cells using CIBERSORTx (28) with scRNA-Seq–derived signatures, which confirmed the prevalence of iATC cells coexisting with mATC cells in ATC tumors (Figure 3D). In total, we detected iATC cells in 11 of 14 ATC tumors in our in-house cohort. Last, our survival analysis of this cohort showed that patients with ATC who had pure mATC tumor cells had significantly (log-rank test *P* = 0.026) worse survival than did patients with mixtures of both iATC and mATC cells (Figure 3E and Supplemental Table 1).

**Figure 4:**
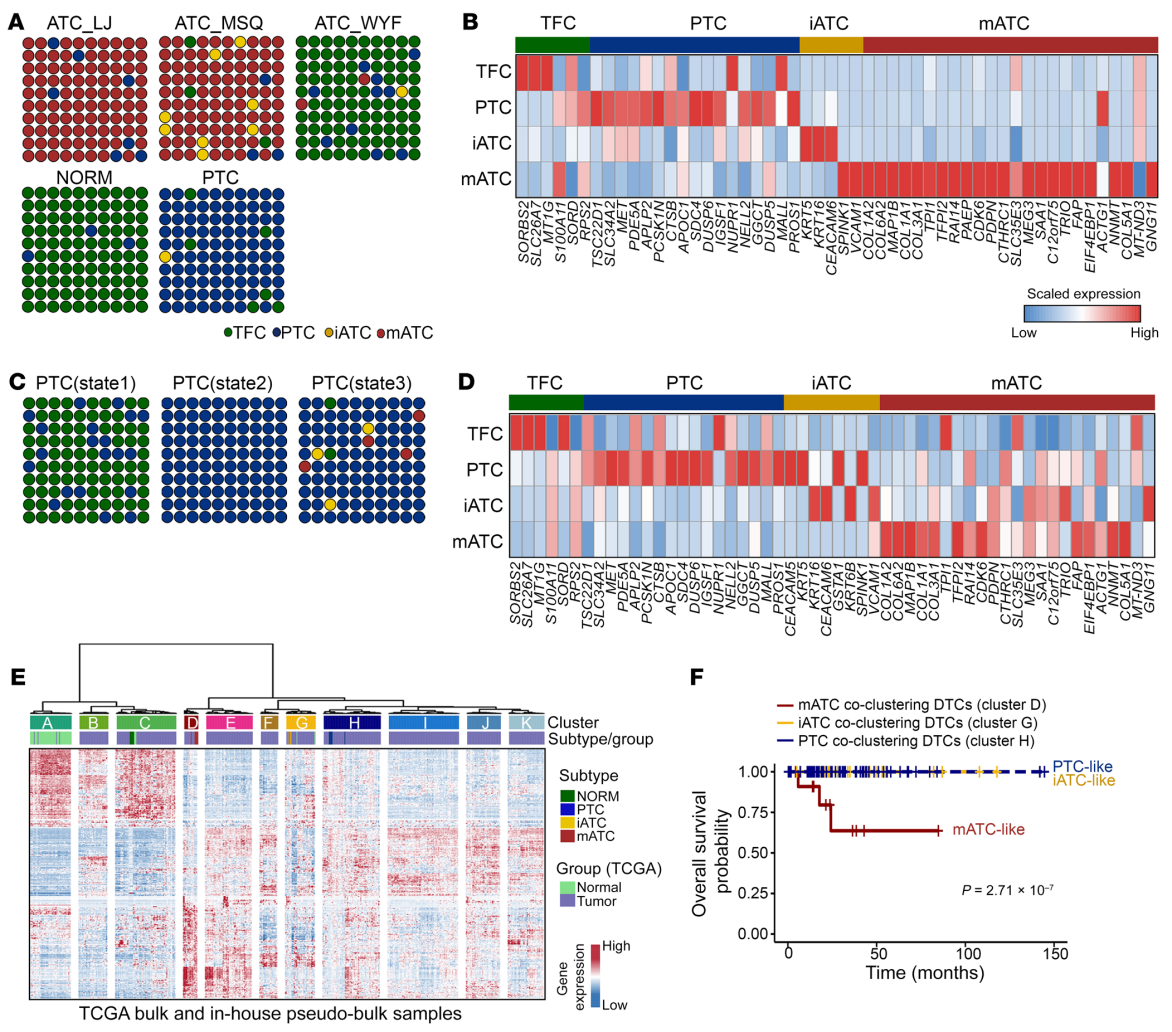
Activation of iATC and mATC signatures in thyroid cancer. (**A** and **C**) Dot plots depict the predicted cell subtype compositions. Normal, PTC, and PTC states are combinations of all cells in each sample type. ATC plots are for individual patients. Data in **A** are from Luo et al. (29), and data in **C** are from Pu et al. (30). (**B** and **D**) Heatmaps showing scaled expression levels of the 59-gene signature with detection in 2 published data sets. (**E**) Coclustering of TCGA bulk RNA-Seq data from Nishant et al. (23), with in-house pseudobulk analysis of scRNA-Seq data based on a union of TFC and tumor-specific genes (Supplemental Table 3). (**F**) Kaplan-Meier plot of overall survival of DTC patients with partial activation of ATC and PTC phenotypes. *P* values were determined by 2-sided log-rank test. +, censored observations.

Next, we sought to gain new insights from previous studies by extending our analysis to published transcriptomic data sets. We identified 1 ATC data set from Luo et al. (29) that included scRNA-Seq data on 3 ATC samples, 6 PTC samples, and 1 normal sample, however the patient survival information was missing. We checked expression levels of the 59-gene predictor and performed subtype prediction with scTypeTC (Figure 4, A and B). Our results revealed that normal samples had mostly TFCs intermixed with approximately 3% PTC cells, whereas the PTC samples consisted mostly of PTC cells intermixed with approximately 1% iATC cells. As expected, the ATC tumors consisted mostly of mATC cells, except for 1 sample (ATC_WYF), which harbored many TFCs that overexpressed thyroid differentiation genes. Similar to our in-house cohort, we detected a high frequency (2 of 3 tumors) of ATC tumors harboring iATC cells. Our analysis confirmed that iATC cells in this cohort activated inflammatory genes such as *CEACAM6*, while mATC cells activated multiple mesenchymal and collagen genes such as *COL1/-3/-5/-6*. A second data set published by Pu et al. (30) included scRNA-Seq data on 6 PTC samples and 5 normal samples, in which the authors classified TFC and PTC cells into 3 developmental states (states 1, 2, and 3) that were associated with PTC progression. Our prediction results showed that state 1 cells comprised mostly TFCs intermixed with some PTC cells and state 2 cells were purely PTC cells, whereas state 3 cells included a mixture of mostly PTC cells and small fractions (3%–4%) of iATC and mATC cells (Figure 4C). Of note, gene expression analysis showed minimal activation of ATC signatures in the predicted iATC and mATC cells (Figure 4D), which was expected because these tumors retained the PTC diagnosis. To investigate the clinical relevance of the partial activation of ATC signatures in the context of differentiated forms of thyroid cancer, we analyzed The Cancer Genome Atlas (TCGA) data set from Nishant et al. (23) that had bulk RNA-Seq data on 567 DTCs and patient survival data. To perform coclustering analysis of these DTCs with our in-house data, we derived pseudobulk transcriptomes of each subtype from scRNA-Seq data on individual patients. In total, we found that approximately 2% of DTC tumors expressing partial mATC signatures were coclustered with mATCs and approximately 6% of DTC tumors expressing partial iATC signatures were coclustered with iATCs (Figure 4E). Interestingly, DTC tumors with partial mATC signatures resulted in significantly worse survival, whereas DTC tumors with partial-iATC signatures did not significantly alter survival outcomes (Figure 4F). We reasoned that the presence of iATC cells may not necessarily affect survival outcomes before the tumor transformed into mATC, as these tumors remained as DTCs at the time of tissue collection.

In summary, we classified ATC tumor cells into 2 major subtypes — mATC and iATC — using the integrative machine-learning and clustering tool scTypeTC. The mATC cells were characterized by a gain of mesenchymal phenotypes and enrichment of collagen-remodeling gene programs. The iATC cells overexpressed neutrophil-related inflammatory pathway genes. Both mATC and iATC cells were dedifferentiated, resulting in a loss of thyroid function gene expression.

### Distinct gene modules drive the continuous progression of thyroid cancer.

**Figure 5:**
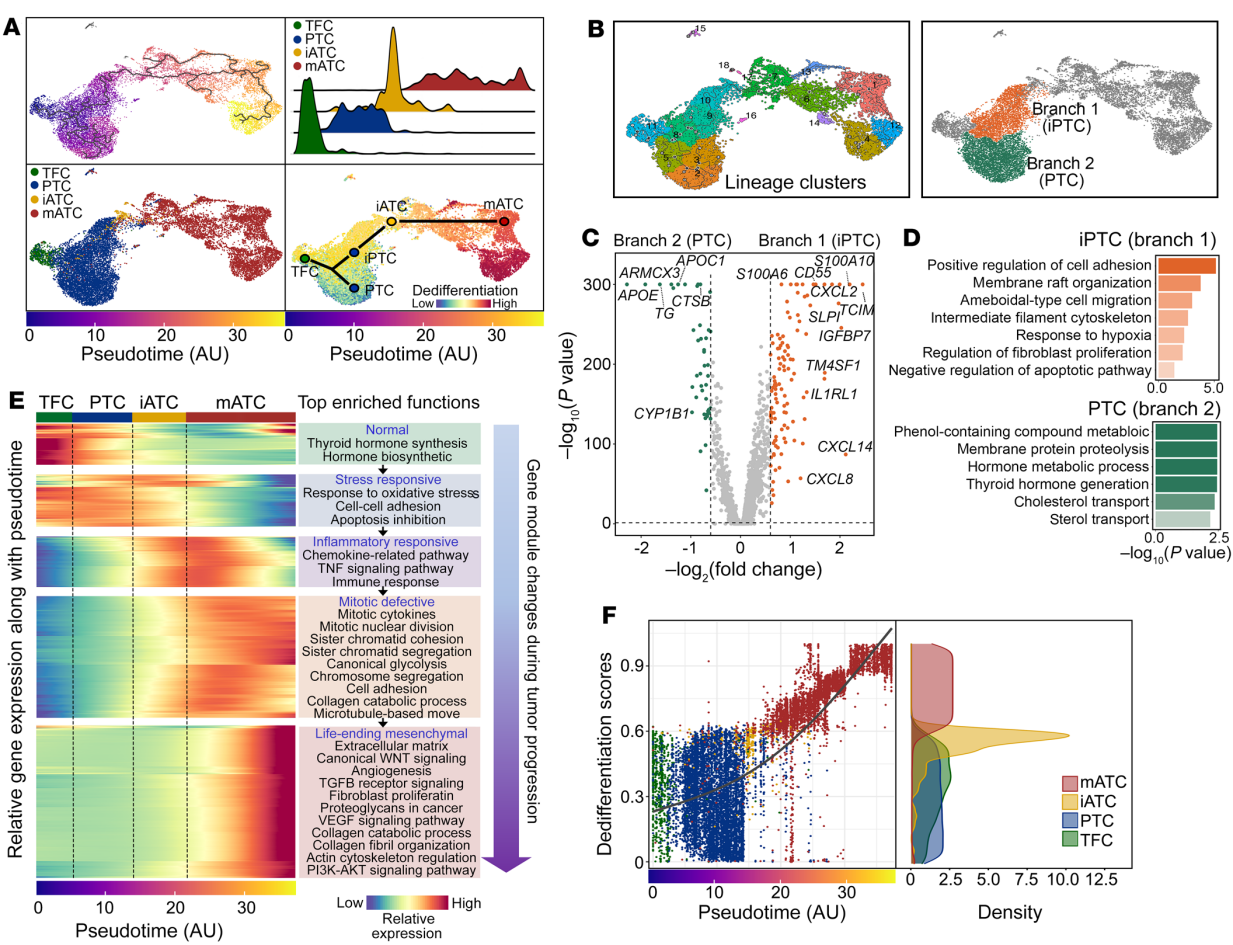
Single-cell transcriptional trajectory during anaplastic transformation in thyroid cancer. (**A**) UMAP projections of single epithelial cells along inferred trajectory by monocle3. Cells are colored by pseudotime (top left), epithelial cell subtypes (bottom left), and dedifferentiation scores inferred by CytoTRACE (bottom right). Top right: Ridge plot of densities of epithelial cell numbers over pseudotime. (**B**) Identification of 2 PTC branches through clustering and trajectory lineages. (**C**) Differential gene expression between 2 PTC branches. *P* values were determined by Wilcoxon test. (**D**) Gene set enrichment comparisons between 2 PTC branches. (**E**) Heatmap of the expression of genes with significant temporal changes. (**F**) Scatterplot of epithelial cells. Right panel shows a density plot of the dedifferentiation scores. AU, artificial unit of pseudotime.

To investigate the temporal dynamics of anaplastic transformation in thyroid cancer, we performed single-cell pseudotime analysis of all epithelial cells in our data set using monocle3 (31). Our analysis revealed a trajectory that was occupied by single cells in pseudotemporal order from normal TFCs to PTCs to iATCs and finally to mATCs (Figure 5A). Notably, the trajectory showed a branching separation of PTCs (Figure 5B). We grouped PTC cells into progressive and nonprogressive branches (Figure 5B) accordingly to compare their transcriptomic profiles. Our analysis showed that the progressive branch was populated by inflammatory PTCs (iPTCs) that overexpressed multiple inflammatory pathway genes such as *CXCL2*, *CXCL8*, *CXCL14*, *S100A6*, *S100A10*, and *IL1R1*, whereas the nonprogressive branch had classical PTC cells that overexpressed metabolic pathways and thyroid differentiation genes (Figure 5, C and D).

We next studied the sequential transcriptional reprogramming activities by performing temporal gene module enrichment and differential gene expression analyses. Our results revealed a stepwise activation of distinct gene modules along different stages of cancer progression, producing a transcriptional reprogramming continuum of anaplastic transformation. This continuum started from the normal functioning states of TFCs, to stress-responsive and metabolic/catabolic deregulatory states of PTC cells, followed by an inflammatory activation state of ATC cells (iATC cells) that further drove cells into a defective mitotic state, and finally, transformation of ATC cells into the mesenchymal state (mATC cells) Figure 5E and Supplemental Table 5). Moreover, a portion of mATC reprogramming events (i.e., overactivation of mitotic programs) were initiated at the later stage of iATCs. This finding suggests that inflammation may induce the formation of a reservoir of different cell states, in which iATC cells act as primers that initiate the anaplastic transformation process.

**Figure 6:**
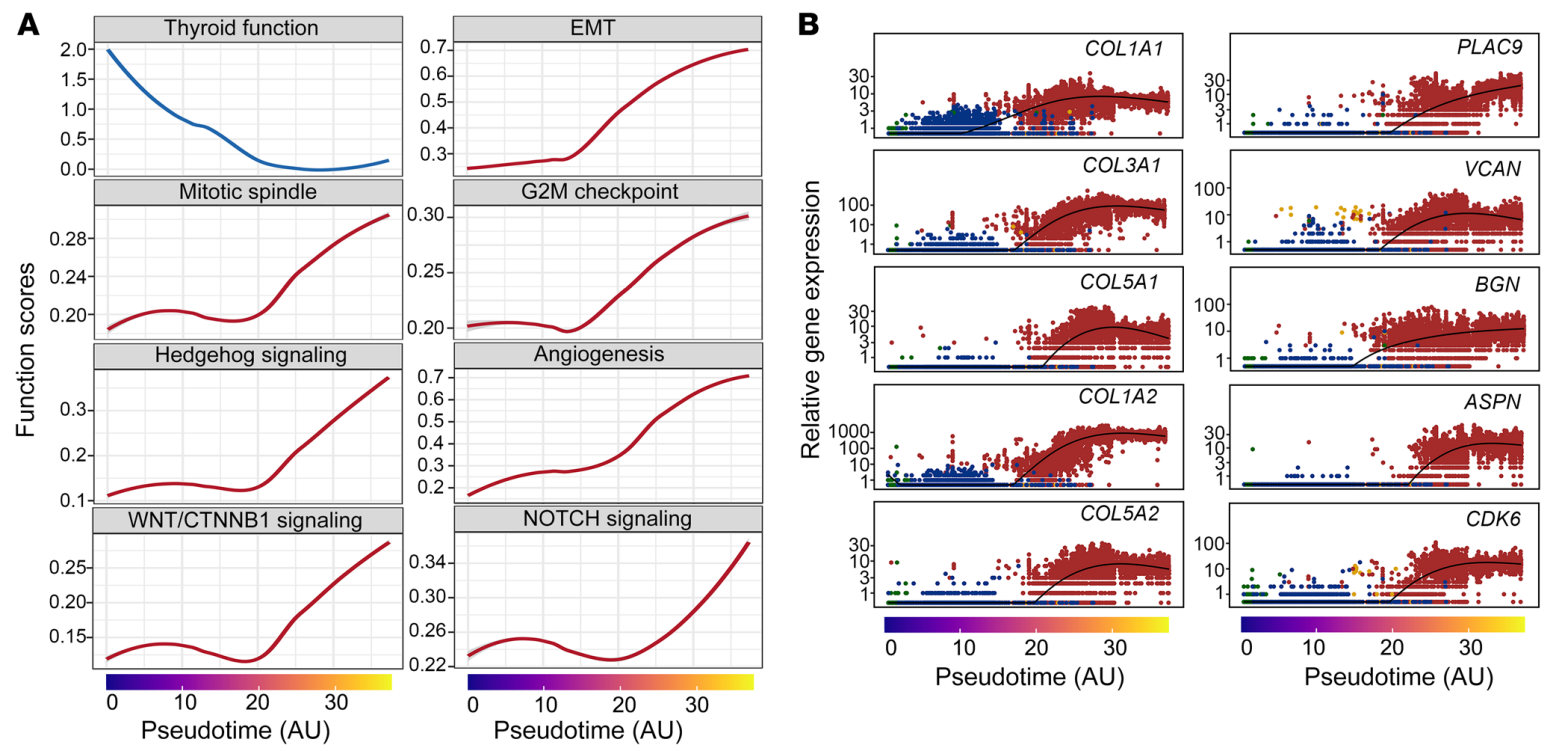
Transcriptional changes underlying anaplastic transformation in thyroid cancer. (**A**) Line plots of the top transcriptional programs. (**B**) Scatter plots of the top genes that were significantly increased along the pseudotime.

Furthermore, we performed dedifferentiation analysis using CytoTRACE (32) to order cells according to their dedifferentiation states (Figure 5F). As expected, our results showed that TFCs and most PTC cells remained differentiated and that iATC cells were moderately dedifferentiated, whereas mATC cells were highly dedifferentiated. Of note, although lower than the scores for both iATC and mATC cells, the dedifferentiation scores for iPTC cells were slightly higher than those for classical PTCs (Figure 5A, bottom right). Consistent with this dedifferentiation gradient, thyroid function scores decreased over pseudotime, and other programs associated with more malignant phenotypes increased, such as EMT, angiogenesis, and NOTCH signaling (Figure 6A).

To identify individual genes that contribute to ATC progression, we examined top genes that were significantly overexpressed along the temporal order (Figure 6B). Interestingly, 5 of the top 10 were collagen genes (i.e., *COL1A1*, *COL1A2*, *COL3A1*, *COL5A1*, *COL5A2*) and 4 were collagen-interacting receptor genes (i.e., *PLAC9*, *ASPN*, *VCAN*, *BGN*), indicating the important roles of ECM factors in promoting ATC aggressiveness. The cyclin-dependent kinase gene *CDK6* was the only noncollagen gene among the top 10. The genes that became underexpressed were mostly related to basic biological processes such as thyroid hormone secretion (*TG*), protein folding (*CLU*), ion channel regulation (*SPINT2*), epithelial cell differentiation (*EPCAM*, *CD24*, *CLDN2*), and metabolism (*TSTD1*, *MAGST1*, *SERPINA1*, *NPC2*) (Supplemental Figure 5), which may represent new therapeutic opportunities that warrant further investigation.

In summary, we projected 4 subtypes of epithelial cells (TFC, PTC, iATC, mATC) onto a branched single-cell development trajectory and defined a transcriptional continuum of anaplastic transformation using single-cell transcriptome data on tumor cells.

### Genetic alterations during thyroid cancer progression.

**Figure 7:**
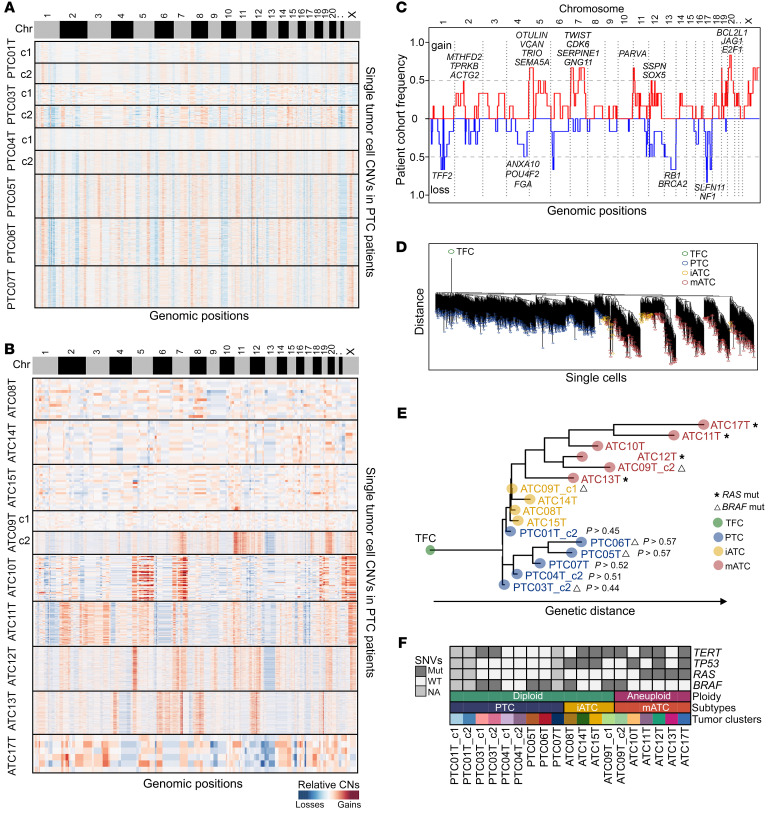
Single-cell CNA and point mutation during thyroid cancer cell progression. (**A** and **B**) Heatmaps of relative copy number ratios of PTC (**A**) and ATC (**B**) tumor cells inferred by CopyKAT. (**C**) Frequency plot of CNAs across patients with mATC. (**D**) N-J tree of single tumor cells with inferred single-cell copy number data. (**E**) M-P tree of tumors cells, rooted to an artificial normal TFC. *P* values were estimated from 1,000 iterations of bootstrap resampling. (**F**) Paneled single nucleotide variants (SNVs) and aneuploidy of epithelial cell clusters for all patients. Mut, mutation.

To investigate the connections between genetic alterations and developmental trajectories, we calculated single-cell DNA copy numbers using CopyKAT (33) and analyzed clinical genotyping results to track evolutionary lineages of these tumors. Our data showed that 67% of ATC tumors harbored aneuploid tumor cells with genome-wide high-magnitude CNAs, whereas 33% of ATC and all PTC tumors were diploids with small, low-magnitude CNAs (Figure 7, A and B). In 1 ATC tumor (ATC09T), we detected a cluster of diploids and a cluster of aneuploids, corresponding to the transcriptional clusters ATC09T_c1 and ATC09T_c2. We collapsed all single-cell CNAs into consensus copy number profiles to detect common CNAs across all patients with ATC, which identified frequent amplifications on chromosomes 2p (*MTHFD2*, *TPRKB*, *ACTG2*), 5 (*OTULIN*, *VCAN*, *TRIO*, *SEMA5A*), 7 (*TWIST*, *CDK6*, *SERPINE1*, *GNG11*), 11p (*PARVA*), 12p (*SOX5*, *SSPN*, *MMP19*), and 20 (*BCL2L1*, *JAG1*, *E2F1*), and frequent deletions of chromosomes 1q (*TFF2*), 13 (*RB1*, *BRCA2*), and 17 (*SLFN1*, *NF1*) (Figure 7C). Intriguingly, amplifications of chromosomes 5, 7, and 20 were associated with overexpression of mesenchymal phenotypes in mATC cells. Moreover, we analyzed the DNA copy number alterations data on DTC samples in TCGA data set (23). In keeping with the observation of aneuploidy in mATC cells, the mATC-like DTC samples also had higher levels of copy number variations (CNVs) compared with the iATC-like DTC samples (Supplemental Figure 6).

To delineate the genomic evolution process during ATC progression, we constructed a neighbor-joining (N-J) tree with inferred low-resolution single-cell CNAs (Figure 7D) and a maximal-parsimony (M-P) tree with pseudobulk CNA data (Figure 7E) as previously described (34). The resulting phylogenetic trees showed that most PTCs and ATCs occupied distinct lineages. A small fraction of PTCs seemed to be closer to the iATC lineage (Figure 7, D and E), indicating potential co-lineaging between a subset of PTCs and iATCs. To investigate the statistical significance, we performed bootstrap resampling of the original single-cell data and rebuilt M-P trees to repeatedly measure the PTC-iATC co-lineages 1,000 times. Notably, our resampling results showed that PTCs were randomly assigned to be adjacent to the iATC lineage (*P* > 0.44–0.57), consistent with our previously published punctuated copy number evolution model in which evolutionary intermediates are rare among diploids (34).

Next, we investigated point mutations in *BRAF*, *RAS*, *TP53*, and *TERT*, which were frequently detected in previous studies and used to stratify patients clinically (15–21). As expected, we observed the *BRAF* *V600E* mutation in 22% of ATC tumors and 43% of PTC tumors, *RAS* mutations in 44% of ATC tumors and 0% of PTC tumors, *TP53* mutations in 67% of ATC tumors and 0% of PTC tumors, and *TERT* mutations in 67% of ATC tumors and 14% of PTC tumors (Figure 7F). The *BRAF* and *RAS* mutations were mutually exclusive, and both *TP53* and *RAS* mutations were only detected in ATC tumors in our cohort. All 4 *RAS*-mutated ATC samples (ATC11T, ATC12T, ATC13T, ATC17T) were classified as the mATC subtype. Of note, the high frequency of *RAS* mutations in mATC samples suggested a possible genetic predisposition for ATC progression rather than an ordered mutation process. Furthermore, we did not observe an association between *BRAF* mutations and PTC bifurcation in the developmental trajectory.

To summarize, the gain of aneuploidy marks a major genetic milestone of ATC progression. The PTC and iATC cells were commonly diploid, representing the early stages, whereas mATC cells often gained aneuploidy, with genome-wide CNAs driving ATC progression to a more lethal stage.

### Remodeling of the tumor immune microenvironment during ATC development.

**Figure 8:**
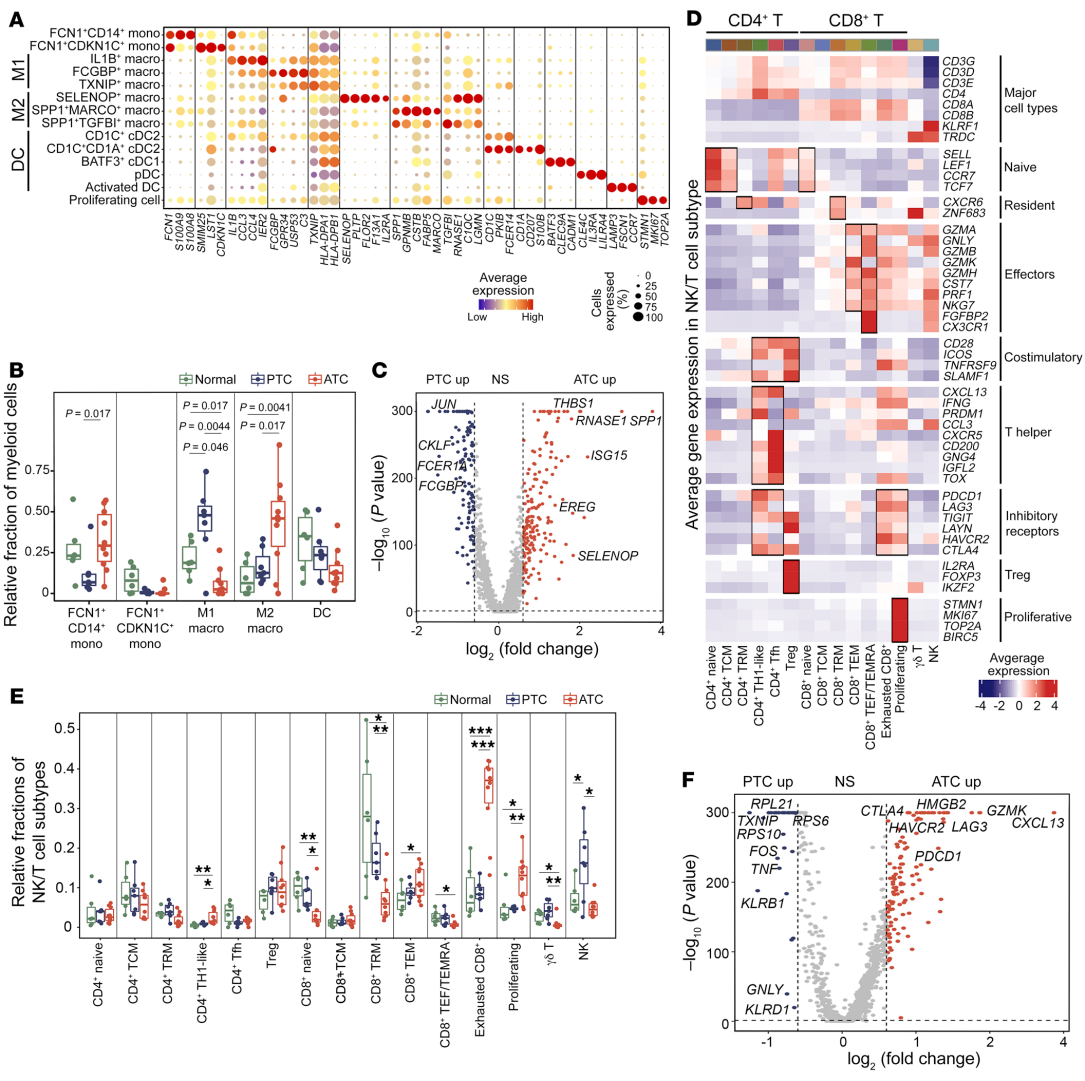
Remodeling of the tumor immune microenvironment during thyroid cancer progression. (**A**) Bubble heatmap of selected marker genes in myeloid cell subpopulations. (**B**) Box plot of relative fractions of major myeloid cell subtypes. *P* values were determined by 2-sided, unpaired Student’s *t* test. (**C**) Volcano plot of differential gene expression analysis of PTC- and ATC-derived macrophages after SoupX. *P* values were determined by Wilcoxon test. up, upregulated. (**D**) Heatmap of gene expression *z* scores for selected marker genes of identified T and NK cell subpopulations. (**E**) Box plot of the relative frequencies of T and NK cell subpopulations. ***P < 0.001, **P < 0.01, and *P < 0.05, by 2-sided, unpaired Student’s *t* test. (**F**) Volcano plot of differential gene expression analysis of PTC- and ATC-derived T and NK cells. *P* values were determined by Wilcoxon test. NS: *P* > 0.05 or log_2_(fold change) <0.6. Macro, macrophages; Mono, monocytes.

In total, we identified 14 myeloid cell subpopulations (Figure 8A and Supplemental Figure 7, A–D, see Supplemental Table 6 for the differentially expressed genes [DEGs]). Comparison analysis revealed a significant increase in M2 macrophages (i.e., SELENOP^+^, SPP1^+^MARCO^+^, and SPP1^+^TGFBI^+^) and decrease in M1 macrophages (i.e., IL1B^+^, FCGBP^+^, and TXNIP^+^) in ATCs compared with PTCs (Figure 8B), suggesting that macrophages were reprogrammed from antitumor functions toward aggressiveness-promoting states. Of note, our study defined a new SELENOP^+^ M2 macrophage that uniquely overexpressed *SELENOP*, which accounts for most of the selenium found in plasma. Differential gene expression analysis between PTC- and ATC-derived macrophages confirmed that both *SELENOP* and *SPP1* were significantly overexpressed in ATC (Figure 8C). The relative cellular fractions of M1 and M2 subpopulations were similar in the iATC and mATC subtypes (Supplemental Figure 7E), however, there were some transcriptional differences imprinted by these 2 subtypes, e.g., mATC-derived macrophages overexpressed M2 marker genes such as *SELENOP*, *FOLR2*, and *F13A1* compared with iATC-derived macrophages (Supplemental Figure 7, F and G).

To investigate the molecular alterations of T cells and NK cells, we classified them into 15 subpopulations (Figure 8, D and E, Supplemental Figure 8, A and B, and Supplemental Table 7). Comparison analysis revealed altered cellular frequencies of several T and NK cell subpopulations in ATC compared with PTC and adjacent normal tissues. The reduced T cell subpopulations in ATC included CD8^+^-naive cells, CD8^+^ tissue-resident memory (TRM) cells, CD8^+^ effector (TEF) or memory effector (TEMRA) T cells, *γδ* T cells, and NK cells (Figure 8E), implying that ATC tumors had less cytotoxic immunity. In contrast, the expanded T cell subpopulations included CD4^+^ Th1-like, CD8^+^ TEM, exhausted CD8^+^, and proliferating exhausted CD8^+^ T cells (Figure 8E), suggesting that most CD8^+^ T cells entered a dysfunctional and exhausted state in ATC. The overall exhausted cells accounted for more than 50% of the T cell populations in ATC tumors, whereas PTC tumors had less than 20% exhausted T cells. Overall cytotoxic cells in ATC accounted for 12% of T cell populations, contrasting with a much higher cytotoxic composition (27%) in PTC tumors (Supplemental Figure 8C). Differential gene expression analysis between PTC- and ATC-derived T and NK cells validated the overexpression of T cell exhaustion genes such as *PDCD1* and *CTLA4* (Figure 8F), providing evidence for the potential efficacy of checkpoint blockade immunotherapy for patients with ATC. The exhausted expansion and cytotoxic reduction phenotypes were similar between iATC and mATC (Supplemental Figure 8, D and E), however, mATC-derived exhausted T cells had higher exhaustion scores than did other subtypes (Supplemental Figure 8, F and G). Enrichment analysis confirmed that mATC-derived T cells were dysfunctional, with upregulation of programmed cell death 1 (PD-1), programmed death ligand 1 (PD-L1), and T cell exhaustion pathway genes (Supplemental Figure 8, H and I).

In summary, the tumor immune microenvironment underwent drastic reprogramming during ATC progression, where macrophages shifted from the M1 to M2 state, and T cells transitioned from a cytotoxic to an exhausted state.

### Speciation of cancer-associated fibroblasts during ATC progression.

**Figure 9:**
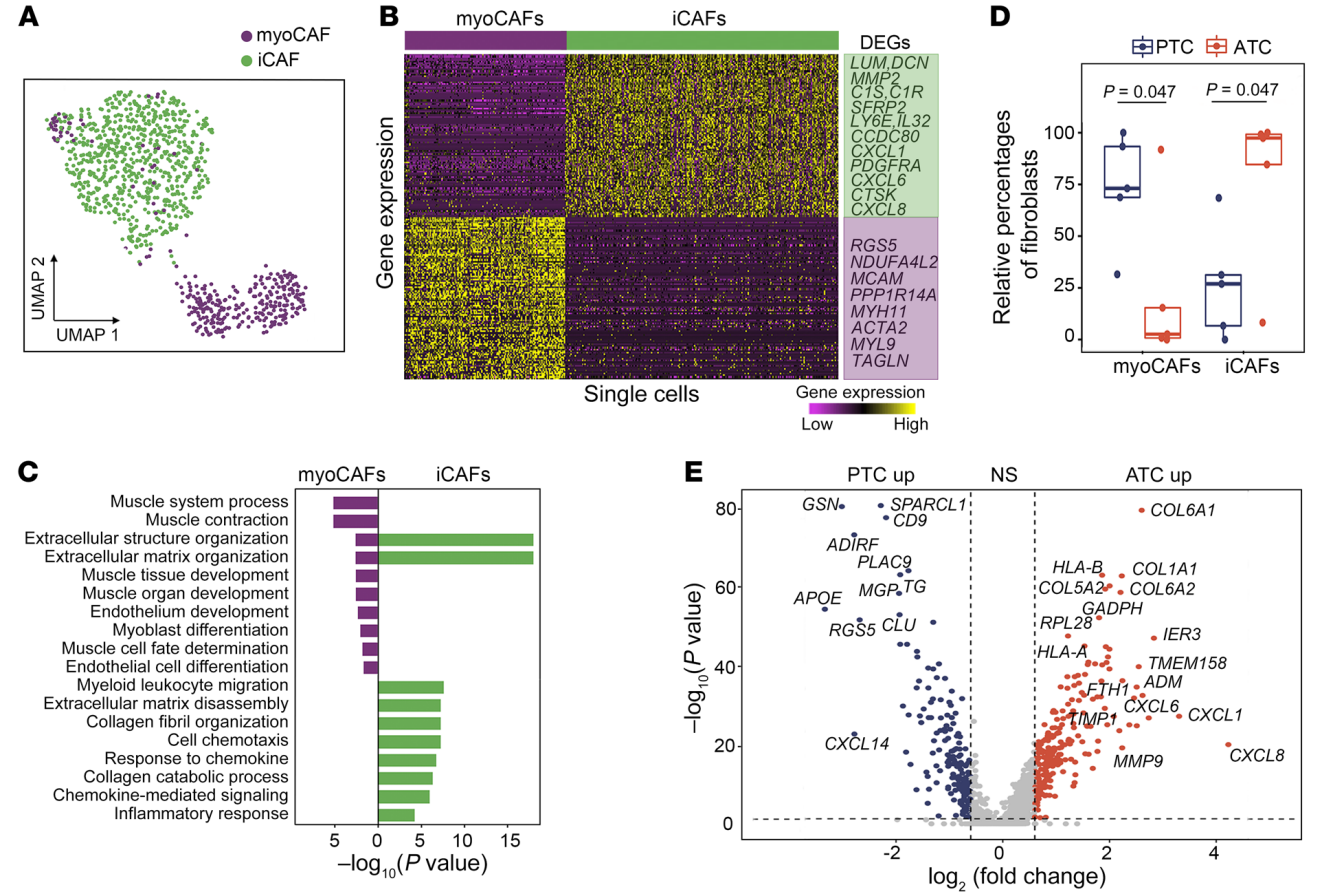
Plasticity of CAFs during ATC progression. (**A**) UMAP projection of CAFs, colored by CAF subpopulations. (**B**) Heatmap of DEGs between myoCAFs and iCAFs. (**C**) Two-sided bar graph showing enriched GO terms in myoCAFs and iCAFs. (**D**) Box plot of relative frequencies of myoCAFs and iCAFs. *P* values were determined by 2-sided Student’s *t* test. The boxes are centered at the median and bounded by Q1 and Q3 quartiles. Upper whiskers indicate the minimum of maximum data and Q3 + 1.5 IQR, and lower whiskers indicate the maximum of minimum data and Q1 – 1.5 IQR. (**E**) Volcano plot of differential gene expression analysis between PTC- and ATC-derived CAFs. *P* values were determined by Wilcoxon test. NS: *P* > 0.05 or log_2_(fold change) <0.6.

We classified all cancer-associated fibroblasts (CAFs) into 2 subtypes: (a) myofibroblastic CAFs (myoCAFs) and (b) inflammatory CAFs (iCAFs) (Figure 9A) that were previously reported (35). The myoCAFs overexpressed myofibroblastic genes such as *ACTA2*, *MCAM*, *MYH11*, and *TAGLN*, whereas iCAFs overexpressed genes involved in inflammation regulation such as *CXCL1*, *CXCL6*, *CXCL8*, *IL32*, *C1S*, and *C1R* (Figure 9B and Supplemental Figure 9A, see Supplemental Table 8 for DEGs). Our gene set variation analysis (GSVA) results confirmed that myoCAFs were enriched for ontologies related to cell contraction functions, whereas iCAFs were enriched for ontologies related to the regulation of inflammation (Figure 9C). Additionally, to identify master regulators of CAF subtypes, we performed transcription factor enrichment analysis using single-cell regulatory network inference and clustering (SCENIC) (36), which detected multiple species-specific transcriptional regulation motifs (Supplemental Figure 9B). Specifically, myoCAFs activated the transcription factors *MEF2C* and *MEF2D*, which regulate muscle contraction functions, and iCAFs activated the transcription factors *STAT1* and *CREB3L1*, which are associated with cytokine and chemokine expression (Supplemental Figure 9C).

Next, we compared the relative cellular frequencies of myoCAFs and iCAFs in ATC versus PTC. Our results showed that iCAFs were mainly detected in ATC (95%), whereas myoCAFs were the major CAF population in PTC (75%) (Figure 9D). Differential gene expression analysis between PTC- and ATC-derived CAFs confirmed overexpression of the iCAF signature in ATC and of the myoCAF signature in PTC (Figure 9E). Furthermore, our data revealed that mATC-derived iCAFs had significant overexpression of cytokines and chemokines, including *CXCL1*, *CXCL3*, *CXCL6*, *CXCL8*, *IL6*, *IL24*, and *IFI27* (Supplemental Figure 9D), as well as higher inflammation scores when compared with iATC-derived iCAFs (Supplemental Figure 9E).

To summarize, we detected 2 subtypes of CAFs (i.e., myoCAFs and iCAFs) in thyroid cancer. The ATC-derived CAFs were mostly iCAFs, whereas the PTC-derived CAFs were mostly myoCAFs. In addition, mATC-derived iCAFs had much higher inflammation scores than did iATC-derived CAFs in ATC tumors.

### Mesenchymal cell types as the hub of cell-cell interactions in ATC.

**Figure 10:**
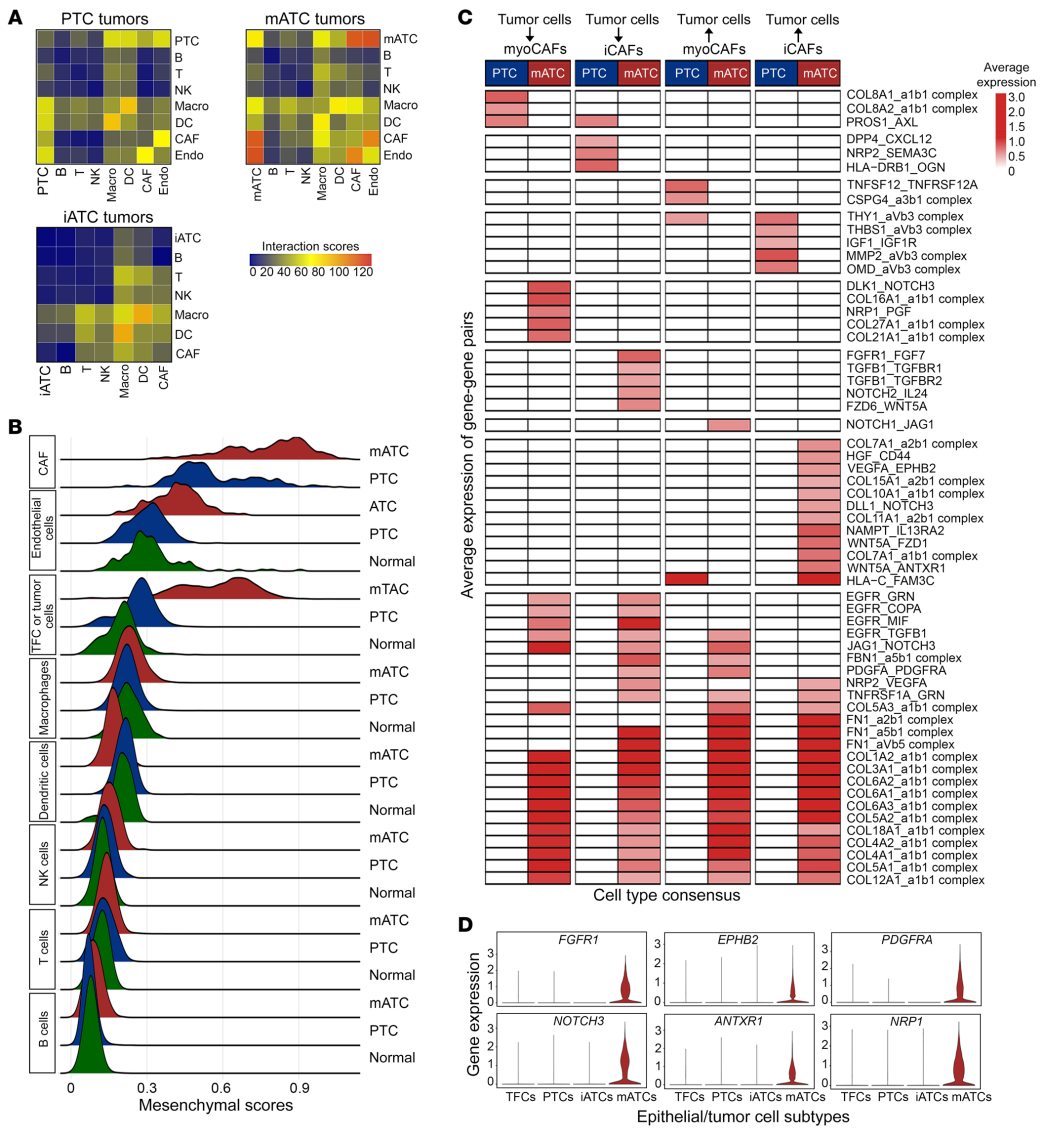
Dynamic changes in cell-cell interactions during ATC progression. (**A**) Heatmaps of significant interaction pairs (*P* < 0.05, by 1-sided permutation test) between major cell types in PTC, iATC, and mATC. (**B**) Ridgeline plots of mesenchymal scores for major cell types in mATC (red), PTC (blue), and normal tissues (green) after SoupX. (**C**) Heatmap showing average expression (avg.exp) levels of uniquely significant ligand-receptor pairs between tumor cells and CAFs in PTC and mATC . The blanks represent nonsignificant interactions. *P* < 0.05, by 1-sided permutation test; mean expression ≥0.5. (**D**) Violin plots of selected receptors in mATC cells contributing to the interaction between tumor cells and CAFs in ATC samples.

We investigated dynamic changes of the cell-cell interaction network in the TME during ATC progression using CellPhoneDB (37). Our results showed that receptor-ligand interactions were mostly detected between tumor cells, CAFs, myeloid cells, and endothelial cells. We observed that mATC tumors had a strong cellular communication network compared with PTC tumors (Figure 10A). Of note, we detected approximately 70 interactions within mATC-mATC cellular pairs, in contrast to the approximately 30 interactions within PTC-PTC cellular pairs, suggesting that mATC tumor cells had stronger self-sufficiency in cellular communication. Intriguingly, the cell types involved in cellular interactions tended to have stronger mesenchymal phenotypes (Figure 10B), among which CAFs had the highest mesenchymal scores, followed by endothelial cells, mATC tumor cells, and macrophages. The mesenchymal scores for all cell types were calculated after the removal of ambient RNAs with SoupX (27). The cell-cell communication in iATC tumors was mostly detected among immune cell types such as macrophages, T cells, and B cells, contrasting with the cell-cell signaling between mesenchymal cell types in mATC tumors (Figure 10A).

In mATC tumors, we observed substantial activation of many collagen and receptor pairs such as collagen families 1, 3, 4, 5, 6, 8, and 12 with the α1β1 complex, facilitating interactions between mATC cells and CAFs (Figure 10C). These collagens were highly expressed in mATC cells, thus promoting tumor and endothelial cell interactions (Supplemental Figure 10). In comparison, only collagen family 8 was uniquely involved in interactions between tumor cells and CAFs in PTC. Additionally, we observed that mATC cells overexpressed receptors such as *FGFR1*, *EPHB2*, *PDGFRA*, *NOTCH3*, *ANTXR1*, and *NRP1*, which could receive signals from CAFs and thereby promote tumor cell proliferation and aggressiveness (Figure 10D). We also detected several significant receptor-ligand interactions between other cell pairs including M2 macrophage–CAF, M2 macrophage–endothelial cell (Supplemental Figure 11A), M2 macrophage–exhausted CD4^+^ T cell, and M2 macrophage–exhausted CD8^+^ T cell (Supplemental Figure 11B), all of which were mostly driven by noncollagen factors.

To summarize, our data showed that mesenchymal cell types, including mATC tumor cells, CAFs, endothelial cells, and M2 macrophages served as a cellular communication hub in the ATC microenvironment. These cellular interactions were strengthened in mATC tumors as a result of ECM remodeling and mesenchymal phenotype switching.

### An anaplastic transformation spectrum in thyroid cancer.

**Figure 11:**
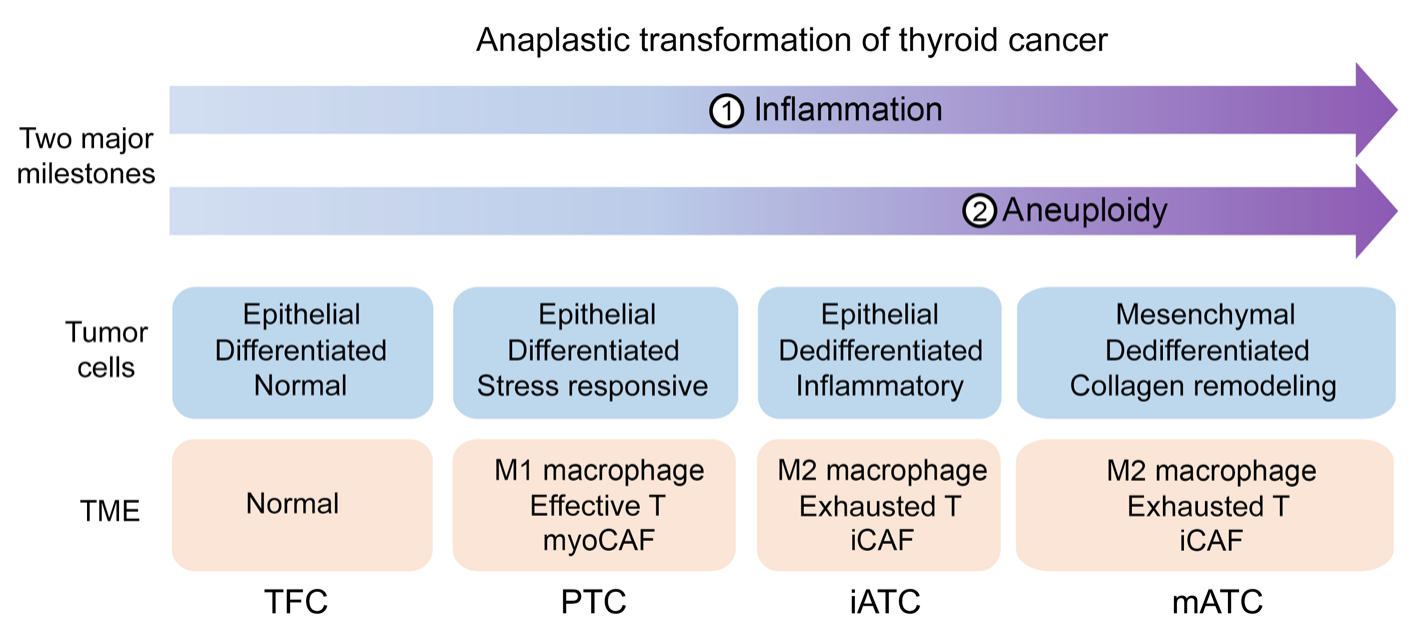
Schematic of anaplastic transformation in thyroid cancer. Molecular characteristics and different transcriptional types of cancer cells during anaplastic thyroid cancer progression.

Our analysis identified 2 key milestones of ATC progression (Figure 11), which had distinct molecular features related to ATC progression: (a) a diploid stage, dominated by diploid iATC tumor cells with inflammatory phenotypes, and (b) an aneuploid stage, dominated by aneuploid mATC cells that had strong mesenchymal and excessive ECM remodeling phenotypes. The aberrant mitosis-related pathways were activated in the late iATC stage and were suspected of being associated with the gain of aneuploidy in mATC cells. Of note, we observed frequent *RAS* mutations in aneuploid mATC tumors, which does not necessarily indicate that these mutations occurred in the later stage of cancer progression. These observations are consistent with our previous study, which showed that *RAS* mutations were associated with more aggressive phenotypes in ATC. In parallel with tumor cell plasticity, myeloid cells reprogrammed from an M1 tumor suppression state to an M2 tumor promotion state. T cells shifted from a cytotoxic to an exhausted state, which we believe highlights the potential of reactivating T cells as a new approach to treating patients with ATC. Taken together, we propose a unified anaplastic transformation model in thyroid cancer, in which PTC cells evolve from normal TFCs by activating stress-responsive and metabolic and catabolic pathways, followed by activation of inflammatory pathways by iATC cells and then deregulation of mitotic pathways to gain aneuploidy, and last, conferring extreme lethality through the overproduction of ECM factors, thereby driving the progression of tumors to terminal stages.

## Discussion

ATC is a highly lethal cancer type that, for decades, has lacked new effective treatment options for patients. Therefore, it remains critical to understand the underpinnings of its evolutionary process from indolent, differentiated forms to aggressive, dedifferentiated forms. In this study, we propose an anaplastic transformation model that involves the sequential reprogramming of tumor cells, the reconstitution of stromal and immune microenvironments, and the acquisition of genome aneuploidy.

Our data show that, during the terminal stages of ATC progression, mATC tumor cells excessively overproduce collagen family members, consistent with previous studies that demonstrated an association between collagens and more malignant phenotypes such as angiogenesis and metastasis (38–41). Our findings, from the transcriptomic perspective, suggest that these collagens are highly active in promoting cellular interactions by binding to their receptors expressed by other types of mesenchymal cells.

Another critical finding of this study was the identification of iATC cells that may have eluded notice in previous microscopic or bulk genomic studies. Although these iATC cells have diploid genomes similar to those of PTC cells, they have more distinct transcriptional programs than do classical PTCs. Specifically, iATCs exhibit a loss of thyroid differentiation markers and do not overexpress genes involved in metabolic/catabolic pathways that are common in PTCs (23). We believe these results add new details of the anaplastic transformation spectrum, filling the gap of the ATC transformation continuum.

Consistent with previous studies suggesting that aneuploid tumor cells could survive by escaping mitotic checkpoints (42), our analysis revealed multiple mitotic pathways that are dysregulated in iATC cells before they transition into an aneuploid stage of excessive collagen production (mATC), suggesting that defective mitotic pathways may play critical roles in reaching the second milestone, i.e., aneuploidy of ATC progression.

Future studies will be needed to develop an experimental system to model the anaplastic transformation process and validate the functional roles of iATC and mATC cells and cell-cell signaling between different cell types in the TME during ATC progression.

Our results provide guidance on how to evaluate the risk of thyroid cancer and better manage this disease based on the patient’s mutational and transcriptional profiles. For instance, the presence of *RAS* mutations and inflammation may suggest a higher risk of ATC transformation, whereas overproduction of collagens may indicate a life-ending stage. These findings also provide evidence that will aid in the development of new therapeutic options for patients with ATC, such as targeting inflammation, mitotic checkpoints, collagens, macrophages, and T cells. Taken together, our expanded knowledge of the anaplastic transformation process sheds light on new therapeutic strategies to improve survival rates and quality of life of patients with ATC.

## Methods

### Human tissues and cell lines.

Fresh thyroid tumors and adjacent normal tissues were obtained from patients with cancer at the UT MD Anderson Cancer Center. The classification of cancer types was determined by histopathological evaluation of H&E-stained tissue sections. Adjacent normal tissues were collected more than 2 cm away from the tumors, except for the NORM19 sample, which was collected from a patient with thymic cancer. All tumor tissues were collected from untreated patients except PTC03 (5 months of treatment with dabrafenib/trametinib) and ATC08 (3 months of treatment with atezolizumab/vemurafenib/cobimetinib). The 9 patient-derived ATC cell lines were obtained through primary culturing of human tumor cell suspensions as previously reported (28).

### Human tissue staining.

All tumor tissue staining experiments were performed at the MD Anderson Cancer Center Research Histology Core Laboratory (28). Tumors were fixed in 10% neutral buffered formalin, paraffin embedded, processed into a 5 μm thick section/slide, and stained with H&E or various antibodies for IHC. For IHC, the slides were deparaffinized, treated with antigen retrieval buffer, blocked with Sniper (catalog BS966, Biocare Medical), stained with primary and the corresponding secondary antibodies, visualized by diaminobenzidine, and counterstained with hematoxylin. The primary antibodies used were as follows: human-specific cytokeratin (CK) 8/18 (catalog M3652, Dako), collagen VI (catalog ab182744, Abcam), thyroglobulin (TG) (catalog BSB 2767, BioSB), CEACAM5/6 (catalog ab22705, Abcam), paired box gene 8 (PAX8) (catalog 379, Biocare), and thyroid transcription factor 1 (TTF-1) (catalog IS05630-2, Dako). Slides were then examined microscopically by a head and neck pathologist using a BX41 Olympus microscope and an Aperio (Leica Biosystems) digital image scanner. The morphology, degree of differentiation (growth pattern, cytologic features, formation of keratin), and extent of inflammation were evaluated from H&E staining results. The IHC staining intensity was graded according to 3 levels: weak, intermediate, and strong.

### scRNA-Seq of human tissues.

We prepared single-cell suspensions from fresh tissues collected from patients with thyroid cancer who had undergone surgery. We minced tissues into 1 mm^3^ pieces in a 10 cm dish with 5–10 mL dissociation solution and then transferred them into a 50 mL conical tube containing 30 mL dissociation solution for dissociation at 37°C in a rotating hybridization oven for 15 minutes to 1 hour. Then, we centrifuged suspensions at 450*g* for 5 minutes to remove supernatant and resuspended the pellets in 5 mL trypsin (25053CI, Corning), followed by incubation at 37°C in a rotating hybridization oven for 5 minutes. We neutralized trypsin with 10 mL DMEM (D5796, MilliporeSigma) containing 10% FBS (F0926, MilliporeSigma). Next, we passed trypsinized suspensions through a 70 mm strainer using a syringe plunger flange to grind the leftover unfiltered tissue and centrifuged the flow-through at 450*g* for 5 minutes to collect the pellets. In cases in which RBCs were present in the pellet, we resuspended the pellets with 10–20 mL 1× MACS RBC lysis buffer (1:10 dilution of 10× MACS RBC lysis buffer, 130-094-183, Miltenyi Biotec) into MilliQ H_2_O to destroy RBCs at room temperature for 10 minutes and applied 10–20 mL DMEM to stop lysis, followed by centrifugation at 450*g* for 5 minutes. We washed the cell pellets with 10 mL 4°C DMEM 3–5 times depending on the number of cells. After centrifugation at 450*g* for 5 minutes, cells were resuspended in cold PBS (D8537, MilliporeSigma) plus 0.04% BSA solution (AM2616, Ambion) and passed through a 40 μm Flowmi filter (h13680-0040, Bel-Art). To make the dissociation solution, collagenase A (11088793001, MilliporeSigma) was dissolved in 75% v/v DMEM F12/HEPES media (113300, Gibco, Thermo Fisher Scientific) and 25% v/v BSA fraction V (15260037, Gibco, Thermo Fisher Scientific) to achieve a final concentration of 1 mg/mL. Finally, single-cell suspensions were sent to a 10X Genomics Chromium system (3′ protocol PN-120237) using V3 chemistry. We sequenced the final libraries on a Novo-Seq 6000 (Illumina), processed raw data into FASTQ files, mapped them to hg20, and summarized the unique molecular identifier (UMI) counts into an expression matrix using CellRanger.

### scRNA-Seq data processing.

Doublets were removed from each sample using the R package DoubletFinder (43) with a loose assumption (i.e., 3% doublet rate). Single cells that had fewer than 200 genes or more than 6,000 genes detected were removed, which filtered out ruptured cells and potential non-singlet cells, respectively. Single cells were required to have more than 1,000 UMIs per cell to illuminate low-depth data. Given the common problem of low viability when single cells are isolated from ATC tumor tissues, cells that had 30% or greater of fractions of mitochondrial expression were filtered out to remove dying cells. In total, approximately 70% single cells were retained for downstream analysis. We ran SoupX (version 1.6.1) (27) on all samples using default parameters to remove ambient RNAs.

### Copy number calculation from scRNA-Seq data.

Single-cell copy numbers were calculated from scRNA-Seq data using R package CopyKAT with default parameters (33). Cells with genome-wide CNAs were predicted as aneuploids, or as diploids if they only had a few or low-magnitude CNAs. Cluster consensus CNA profiles were calculated as the averages of all cells in same clusters. Averages were taken by each genomic position across all cells in the cluster.

### Major cell type detection from scRNA-Seq data.

Automated cell-type prediction was combined with manual annotation to precisely determine cell types of single cells. R package SingleR (24) was applied to predict cell types using the built-in Human Primary Cell Atlas data as a reference. We labeled predicted cell types with less than 0.5% of total cell numbers or fewer than 2 cells as not defined. Clustering of all cells was performed for each patient, and each cluster was annotated using the best vote rule (i.e., assign cell types by clusters with the most frequent prediction results). Finally, the prediction was curated using established markers: T cells (CD4/CD8A/CD8B), B cells (CD19/CD20) or other immune cells (CD45), epithelial cells (EPCAM/KRT18/KRT8) and endothelial cells (PECAM1/CD34). Since both tumor and normal follicular cells have epithelial origins, additional principles were used to separate them. First, epithelial cells with large-scale, genome-wide CNAs were claimed as tumor cells. Then, epithelial cells expressing thyroid cancer genes were labeled as tumor cells as well. Last, epithelial cells that did not have many CNAs and expressed thyroid differentiation genes were labeled as normal epithelial cells.

### Machine learning of epithelial cell subtypes.

R package glmnet (26) was used to perform machine learning to classify epithelial cell subtypes including both normal follicular and tumor cells by fitting a generalized linear model with lasso-penalized maximum likelihood. To train the model, we first derived a list of DEGs [adjusted *P* < 0.001, log_2_(fold change)≥1] in epithelial cells by comparing the transcriptomes of epithelial cells with other cell types within each patient sample to mitigate patient-by-patient batch effects using R package Seurat. Next, we pooled all epithelial cell DEGs for all patients to generate a union of DEGs (*n* = 832 genes) and perform unsupervised hierarchical clustering of the consensus epithelial cell transcriptomes for all patients with complete linkage and Pearson correlation distance using R package pheatmap, which led to the identification of 5 major clusters of epithelial cells. One cluster with low data quality was filtered out because of missing expression of multiple housekeeping genes. We then used these unsupervised clustering labels and the DEGs across these unsupervised clusters to build a pseudotraining data set to train glmnet models. To select a subset of gene variables that could powerfully predict the subtypes of individual cells, we fitted a multinomial lasso model with 1,000 gradient regularization parameters ranging from 0.1 to 1. Genes that had a greater than 50% subtype-specific prediction power and less than 10% nonspecific prediction power were selected as subtype predictors and used to optimize the multinomial lasso models. In total, a unified 59-gene predictor was defined on the basis of 1,000 test runs sampling 30% of the single-cell data with gradient α values ranging from 0 to 1. Finally, these predictor genes were used to predict epithelial subtypes of single cells using the multinomial lasso model by following the best vote principles. Cells with less than a 50% chance of consistent predictions were labeled as undefined.

### Clustering and survival analysis of TCGA thyroid tumor and normal samples.

Coclustering methods were used to estimate the similarity of a large cohort of TCGA thyroid tumor and matched normal samples, with the subtypes defined by our machine-learning approaches. Bulk RNA-Seq expression data were downloaded, together with sample annotations and survival information on patients with thyroid carcinoma (TCGA-THCA) from TCGA website (https://portal.gdc.cancer.gov). To perform the coclustering, we first created pseudobulk gene expression profiles from our single-cell transcriptome data for each sample by calculating the average expression level of each gene in all epithelial cells in individual samples. DEGs between the 4 subtypes of pseudobulk data were calculated [*P* < 0.05 and |log_2_(fold change)| >0.5] using the Wilcoxon rank-sum test. Next, our in-house pseudobulk data were integrated with TCGA data using an empirical Bayesian framework to remove batch effects with the “combat” function (44) in R package sva (45). Next, hierarchical clustering was performed using Euclidean distance and Ward D linkage based on integrated expression profiles of pseudobulk DEGs across the 4 subtypes. Finally, classified all samples were classified into 11 hierarchical clusters using the “cutree” function in R package pheatmap to identify samples that expressed transcriptional programs similar to those of normal, PTC, iATC, and mATC subtypes for further survival analysis. Survival analysis comparing groups was performed using the log-rank test in R package survival (46). Kaplan-Meier survival curves were plotted using R package survminer (47) to show differences in survival time.

### Construction of single-cell trajectories.

R package monocle3 (48) was applied to construct single-cell trajectories from scRNA-Seq data. To run monocle3 analysis, we first processed the raw scRNA-Seq UMI count matrix with R package Seurat (version 4.0). Raw UMI counts were normalized to total UMI counts per cell using the negative binomial regression method, and the top 3,000 highly variable genes were selected using the variance-stabilizing transformation (VST) method implemented in the “SCTransform” function in Seurat. Then, principal components (PCs) were calculated using Seurat “RunPCA” functions, and batch effects were corrected across samples using Harmony (49) with default parameters and 10 iterations. Harmony-adjusted PCs were used to perform uniform manifold approximation and projection (UMAP) analysis using the RunUMAP function in Seurat. Next, the Seurat object was converted into a SingleCellExperiment object, and UMAP embeddings from Seurat were input to run monocle3. We used the “cluster_cells, learn_graph, and order_cells” functions with default parameters (except for cluster_cells, resolution = 0.0006). The TFC region of the graph was marked artificially as the beginning of the trajectory based on our empirical knowledge. Additionally, PTC cells were separated into 2 subpopulations on the basis of the bifurcated lineages, i.e., branch1 (cluster8, cluster9 and cluster10) and branch2 (cluster 2, cluster3 and cluster5). To detect genes that were differentially expressed across the single-cell trajectory, graph autocorrelation analysis was applied using the graph_test based on principal graph tests (Moran’s I). DEGs were selected by q_value == 0 & morans_I > 0.25 and plotted by R package ComplexHeatmap. Finally, the gene ontology (GO) biological process (BP) and Kyoto Encyclopedia of Genes and Genomes (KEGG) pathway enrichment analysis of each gene module was performed using the Database for Annotation, Visualization and Integrated Discovery (DAVID) (https://david.ncifcrf.gov).

### Construction of the N-J tree based on inferred single-cell DNA copy numbers.

R package ape (50) was used to construct N-J trees from inferred single-cell copy number data. To better sample representative cells to construct trees, all cells for tumor clusters with fewer than 100 cells were used. If a tumor cell cluster had more than 100 cells, we randomly downsampled 100 cells. The pairwise Euclidean distance between cells was calculated using the “dist” function in R, and the N-J tree was then built using the “nj” function. The final tree was re-rooted to an artificial normal diploid cell using the interactive “root.phylo” function.

### Construction of the M-P tree with inferred consensus DNA copy numbers.

M-P trees were constructed from the consensus copy number event matrix using R package phangorn (51). Consensus copy number profiles were obtained by taking the averages of all cells within individual tumor cell clusters. Copy number variations (CNVs) on autosomes with log_2_ segment values of 0.06 or greater were defined as copy number “gain,” less than or equal to –0.06 defined as “loss,” and between –0.06 and 0.06 as “neutral.” The final M-P trees were calculated from the tri-event matrices using the parsimony ratchet algorithm, where the branch lengths and ancestral probabilities were calculated using the Acctran algorithm. For better visualization, the tree was re-rooted to an arbitrary diploid node.

To investigate statistical significance of the co-lineaging of PTC clusters with ATC clusters, we bootstrapped 30% of single cells in each cluster and recalculated the tri-event matrices to build resampled M-P trees using the same parameters. Next, the resulting trees were cut at the common ancestor node where individual clusters were separated into major lineages. Last, we examined whether each PTC cluster co-occupied the same major lineages as the ATC clusters. This resampling process was repeated 1,000 times. We subtracted the simulated probabilities of PTC and ATC clusters being coassigned into the same major lineages from 1 as the simulated *P* values. Since iATC clusters were a portion of the ATC samples, we reasoned that the chance of PTC being assigned to iATC lineages was lower than the simulated *P* values.

### Prediction of differentiation states.

R package CytoTRACE (version 0.3.3) was used to predict the differentiation states of epithelial cells in our scRNA-Seq data. The raw count matrix was input to generate the prediction using the CytoTRACE function, and dedifferentiation scores were plotted with the plotCytoTRACE function using UMAP coordinates generated from Seurat.

### Calculation of phenotype scores.

The average expression of thyroid function genes (*TG*, *TPO*, *SLC26A7*, *ID4*, *PAX8*, *IYD*, *ID3*, *TSHR*, *SLC26A4*) in single cells was calculated as thyroid function scores; M1 macrophage–associated genes (*CCL5*, *IL1A*, *IL1B*, *IL18*, *CCR7*, *CD40*, *CD86*, *HLA-DPB1*, *HLA-DPA1*) as M1 phenotype scores; M2 macrophage–associated genes (*CCL13*, *CCL18*, *CCL20*, CD68, *CD276, CD209, CD163*, *CTSA*, *CTSB*, *CTSD*, *FN1*, *LYVE1*, *MMP9*, *MMP14*, *MMP19*, *MSR1*, *MRC1*) (52, 53) as M2 phenotype scores; cytotoxicity-associated genes (*CST7*, *GZMA*, *GZMB*, *IFNG*, *NKG7*, *PRF1*, *GZMK*, *GZMH*, *CCL3*) as cytotoxicity scores; and exhausted marker genes (*CTLA4*, *HAVCR2*, *LAG3*, *PDCD1*, *TIGIT*) (53, 54) as exhaustion scores for single cells. Other function scores were calculated as the mean expression of genes involved in EMT (mesenchymal score), inflammation, mitotic spindle, the G_2_M checkpoint, hedgehog signaling, angiogenesis, WNT/CTNNB1 signaling, and NOTCH signaling, according to the hallmark gene set data obtained from the Molecular Signatures Database (MSigDB) (https://www.gsea-msigdb.org/gsea/msigdb/).

### Reclustering of immune cell and CAF subpopulations.

To identify subpopulations of myeloid cells, T cells, NK cells, and fibroblasts, we reclustered cells of major cell types for all patients. A small population of fibroblasts (23 cells) in normal samples was detected. As an exception, only the subclusters of CAFs in tumor samples were analyzed. To reduce batch effects between patients, the data were integrated using the fast mutual nearest-neighbor correction (fastMNN) algorithm (55) in SeuratWrappers for myeloid cells and CAFs. Because of the different levels of batch effects in T cells and NK cells, canonical correlation analysis (CCA) was applied within the “FindIntegrationAnchors” and “IntegrateData” functions in Seurat for T cells and NK cells. The top 2,000 variable genes in each sample were used as integration features for both the CCA and fastMNN methods. The first 30 fastMNN PCs were sent for UMAP and *t*-distributed stochastic neighbor embedding (*t*SNE) and cluster identification. For subclustering of T cells and NK cells, the first 20 dimensions of the CCA and principal component analysis (PCA) were used for *t*SNE and cluster identification. The UMAP and *t*SNE plots were generated using Seurat’s built-in functions.

### Annotation of immune cell subpopulations.

To annotate individual immune cell subclusters, differential gene expression analysis was performed to detect significantly overexpressed genes in individual subclusters using the Seurat “FindAllMarkers” function. To perform better annotation, loose criteria were set to select more signature genes to characterize subclusters [i.e., expressed in more than 25% in at least 1 population, adjusted *P* < 0.05 by Wilcoxon rank-sum test and log_2_(fold change) >0.25]. Last, the subclusters were annotated based on the overlapping of signature genes with known markers in previous publications.

Specifically, myeloid clusters were annotated into subpopulations using published gene markers (53, 56). The FCN1^+^CD14^+^ monocytes expressed classical monocyte-associated genes including *CD14*, *S100A8*, and *S100A9*. The FCN1^+^CDKN1^+^ monocytes expressed nonclassical markers *CDKN1C*. Macrophages were identified by their high expression levels of *CD68* and *CD163*. The 6 macrophage subpopulations (IL-1B^+^, FCGBP^+^, TXNIP^+^, SELENOP^+^, SPP1^+^MARCO^+^, SPP1^+^TGFBI^+^) were identified on the basis of their signature genes. Additionally, DCs were classified into 5 subpopulations. Conventional type 1 DCs (cDC1) were annotated according to their overexpression of classical cDC1 marker genes (i.e., *BATF3* and *CLEC9A*). Conventional type 2 DCs (cDC2) were annotated according to their overexpression of *CD1C*. Because of the higher intracluster heterogeneity of this cDC2 subpopulation, we further divided it into 2 subclusters (i.e., CD1C^+^ cDC2 and CD1C^+^CD1A^+^ cDC2 subpopulations). Plasmacytoid DCs (pDCs) and activated DCs were annotated according to their overexpression of *IL3RA* and *LAMP3*, respectively. As noted, we also detected some proliferating cells on the basis of their high expression of proliferation genes (*STMN1*, *MKI67*, *TOP2A*, *BIRC5*) and a small fraction of undefined low-quality myeloid cells.

T cell and NK cell subpopulations were classified as follows: naive T cells carried a naive signature (*SELL*, *LEF1*, *CCR7*, and *TCF7*). TCM cells expressed lower levels of naive markers and higher levels of *ANXA1*, *GRP183*, and *IL7R*, consistent with previous studies (57, 58). TRM cells expressed the tissue-resident marker genes *CXCR6* and *ZF683*. Both T follicular helper (Tfh) cells and T helper type 1 (Th1) cells expressed *CXCL13*, *TOX*, *IGFL2*, *CD200*, and *GNG4*. However, Th1-like cells uniquely expressed *IFNG*, *CCL3*, and *PRDM1* compared with Tfh cells, which separated these 2 closely related subtypes in our data. The suppressive CD4^+^ Tregs expressed high levels of costimulatory and immunosuppressive marker genes (*IL2RA*, *FOXP3*, and *IKZF2*). Consistent with a previous study (59), we identified CD8^+^GZMK^+^ T cells as TEM cells, the precursors of cytotoxic T cells, which expressed several cytotoxic genes (*GZMA*, *GZMH*, *GZMK*, *NKG7*, *CST7*). We observed a cluster of CD8^+^ TEF/TEMRA cells, which overexpressed chemokine receptor (*CX3CR1*) and cytotoxic genes (*FGFBP2*, *GNLY*, *GZMH*), but had much lower levels of T cell exhaustion markers and naive markers. The exhausted CD8^+^ cells showed elevated expression of T cell exhaustion marker genes, such as *PDCD1*, *LAG3*, *TIGHT*, *LAYN*, *HAVCR2*, and *CTLA4*. In addition, we detected a cluster of CD8^+^ proliferating T cells that overexpressed both proliferation genes (*STMN1*, *MKI67*, *TOP2A*, *BIRC5*) and inhibitory receptors. Interestingly, most cytotoxic markers were also highly expressed in exhausted CD8^+^ cells and proliferating exhausted CD8^+^ cells, except for *GNLY*, *FGFBP2*, and *CX3CR1*, consistent with previous publications (57, 60, 61). Additionally, γδ T cells and NK cells were identified by their expression of *TRDC*, *CD3E*, and *KLRF1* genes, respectively.

### Differential gene expression analysis.

The “FindMarkers” function in Seurat was used to detect DEGs in a cluster that was compared with other clusters, which included DEGs between 4 epithelial subtypes, DEGs between all PTC- and ATC-derived macrophages, DEGs between all PTC- and ATC-derived T and NK cells, DEGs between all PTC- and ATC-derived CAFs, DEGs between iATC- and mATC-derived macrophages, DEGs between exhausted CD8^+^ T cells in iATCs and mATCs, DEGs between myoCAFs and iCAFs, and DEGs between iCAFs in iATCs and mATC. DEGs were required to be expressed in more than 25% of cells within at least 1 group [adjusted *P* < 0.05 by Wilcoxon rank-sum test and log_2_(fold change) >1]. To define marker genes for 3 tumor cell subtypes, we used normal TFCs were used as a control and required an adjusted *P* value of less than 0.05 and a log_2_(fold change) of greater than 0.58.

### Functional gene set enrichment analysis.

The enrichment analyses of GO BP and KEGG pathways were performed using R package clusterProfiler (version 4.0.5) (62). Ingenuity Pathway Analysis (IPA) was also used to perform pathway enrichment analysis to compare the functional programs of iATC- and mATC-derived exhausted CD8^+^ T cells. Further, we applied GSVA, a nonparametric and unsupervised algorithm, to assess different pathway activities in different subtypes of tumors using R package GSVA (version 1.40.1) with default parameters (except for min.sz = 10, max.sz = 500), followed by differential enrichment score analysis using R package limma.

### Transcription factor activation analysis.

To compare the differences in transcription factors and their target genes between myoCAFs and iCAFs, SCENIC analysis was performed on single cells using R SCENIC package (version 1.2.4) (36) with default parameters (corresponding to RcisTarget version 1.10.0 and AUCell version 1.12.0). Raw UMI count data in the Seurat object were used as input, and the gene regulatory network was identified for individual cells. The regulon group heatmap was plotted using R package pheatmap.

### Cell-cell interaction analysis.

CellPhoneDB (version 2) (37) was used to identify cell-cell interactions between different cell types in PTC, iATC, and mATC tumor samples. Receptor-ligand interactions between specific cell types were identified according to the expression of a receptor in 1 cell type and a ligand in another cell type. Only receptor or ligand genes expressed in more than 25% of the cells in the specific cell types were selected. In addition, the cell-type labels of all cells were randomly permuted 1,000 times to obtain the significant receptor–ligand pairs (*P* < 0.05) between the different cell types by calculating the proportion of the means that were as high as or higher than the actual mean. The interaction scores refer to the total amount of significant receptor-ligand interaction pairs between specific cell types.

### Data and software availability.

The raw sequencing data and gene expression matrix from this study were deposited in the NCBI’s Gene Expression Omnibus (GEO) database (GEO GSE193581). The R package scTypeTC is publicly available on GitHub (https://github.com/gaolabtools/scTypeTC; main branch, latest commit ID: e9a6536). The *Nature Communications* (30) data set was downloaded from GEO (GEO GSE184362). The *Science Advances* (29) data set was downloaded from GEO (GEO GSE210347). TCGA data set (23) was downloaded from TCGA Data Portal (https://tcga-data.nci.nih.gov/tcga/; project ID: TCGA-THCA).

### Statistics.

All statistical analyses and presentation of these analyses were performed using open-sourced R (version 4.1.1). Cell proportion comparisons between 2 groups were performed using an unpaired, 2-tailed Student’s *t* test. To determine statistical significance for gene expression or gene signatures between 2 groups of cells, an unpaired, 2-tailed Wilcoxon rank-sum test with Bonferroni’s correction was applied. The statistical tests used in the figures are specified in the figure legends and in Methods, and statistical significance was set at a *P* value of less than 0.05.

### Study approval.

The collection of tumor tissues from patients with cancer was approved at the UT MD Anderson Cancer Center under IRB protocols (LAB09-0337 and PA19-0402). The IRB protocol (STU00216474) for the genomics analysis of deidentified human tissues was approved at Northwestern University.

## Author contributions

LL led and performed data analysis and wrote the manuscript. JRW obtained clinical samples, analyzed data, reviewed clinical data, and edited the manuscript. LL and JRW were assigned co–first authorship because their contributions were considered equally essential to this project, with LL listed first because of the extensive computational analysis she performed and the heavy dependence on the conclusions drawn from her analysis. YCH, SB, JZL, and TMT performed experiments and edited the manuscript. JY and YY performed data analysis. MH preprocessed all raw sequencing data. CKS, TP, RK, and XZ reviewed the analysis results and edited the manuscript. MDW performed experiments, analyzed clinical data, and edited manuscript. MEC, RD, and NLB reviewed the data analysis results, analyzed clinical samples, and edited the manuscript. JW performed experiments. RN reviewed data analysis. MZ collected clinical samples and reviewed the analysis results. NN generated data, reviewed results, and edited the manuscript. SYL designed experiments, developed clinical protocols, supervised clinical sample collection, reviewed data analysis results, and edited the manuscript. RG supervised the overall study, designed experiments, performed data analysis, and wrote the manuscript.

## Supplementary Material

Supplemental data

Supplemental tables 1-8

## Version 1 04/13/2023

In-Press Preview

## Version 2 06/01/2023

Electronic publication
