# Supplementary material for: Anaplastic transformation in thyroid cancer revealed by single-cell transcriptomics
Source: J Clin Invest. 2023 Jun 1;133(11):e169653. doi: 10.1172/JCI169653 (PMC10231997; doi:10.1172/JCI169653)
Supplement: Supplemental data [file jci-133-169653-s101.pdf]

Supplementary Figures

Anaplastic Transformation in Thyroid Cancer Revealed by Single Cell Transcriptomics

Lina Lu[1,2,#], Jennifer Rui Wang[3,#], Ying C. Henderson[3], Shanshan Bai[4,5], Jie Yang[6], Min Hu[4], Cheng-Kai Shiau[1,2], Timothy Pan[7], Yuanqing Yan[8], Tuan M Tran[4], Jianzhuo Li[4], Rachel Kieser[9,10], Xiao Zhao[4], Jiping Wang[3], Roza Nurieva[11], Michelle D. Williams[12], Maria E. Cabanillas[13], Ramona Dadu[13], Naifa Lamki Busaidy[13], Mark Zafereo[3], Nicholas Navin[4,14,15], Stephen Y. Lai[3,14,16,17,*], Ruli Gao[1,2,4,7,10,*,^]

[1] Department of Biochemistry and Molecular Genetics, Northwestern University, Chicago, IL, USA 60611
[2] Center for Cancer Genomics, Robert H. Lurie Cancer Center, Northwestern University, Chicago, IL, USA 60611
[3] Department of Head and Neck Surgery, The University of Texas MD Anderson Cancer Center, Houston, TX, USA 77030
[4] Department of Genetics, The University of Texas MD Anderson Cancer Center, Houston, TX, USA 77030
[5] Department of Genitourinary Medical Oncology, The University of Texas MD Anderson Cancer Center, Houston, TX, USA 77030
[6] Department of Radiation Oncology, New York University Langone School of Medicine, New York, NY, USA 10016
[7] The Driskill Graduate Program, Northwestern University, Chicago, IL, USA 60611
[8] Department of Surgery, Northwestern University, Chicago, IL, USA 60611
[9] Center for RNA Therapeutics, Department of Cardiovascular Sciences, Houston Methodist Research Institute, Houston, TX, USA 77030
[10] Center for Bioinformatics and Computational Biology, Department of Cardiovascular Sciences, Houston Methodist Research Institute, Houston, TX, USA 77030
[11] Department of Immunology, The University of Texas MD Anderson Cancer Center, Houston, TX, USA 77030
[12] Department of Pathology, The University of Texas MD Anderson Cancer Center, Houston, TX, USA 77030
[13] Department of Endocrine Neoplasia and Hormonal Disorders, The University of Texas MD Anderson Cancer Center, Houston, TX, USA 77030
[14] Graduate School of Biological Sciences, University of Texas, Houston, TX, USA 77030
[15] Department of Bioinformatics and Computational Biology, The University of Texas MD Anderson Cancer Center, Houston, TX, USA
[16] Department of Radiation Oncology, The University of Texas MD Anderson Cancer Center, Houston, TX, USA 77030
[17] Department of Molecular and Cellular Oncology, The University of Texas MD Anderson Cancer Center, Houston, TX, USA 77030

[#] Co-first authors
^ Lead contact
*** Co-senior and co-correspondence:** ruli.gao@northwestern.edu (R.G.); SYLai@mdanderson.org (S.L.)

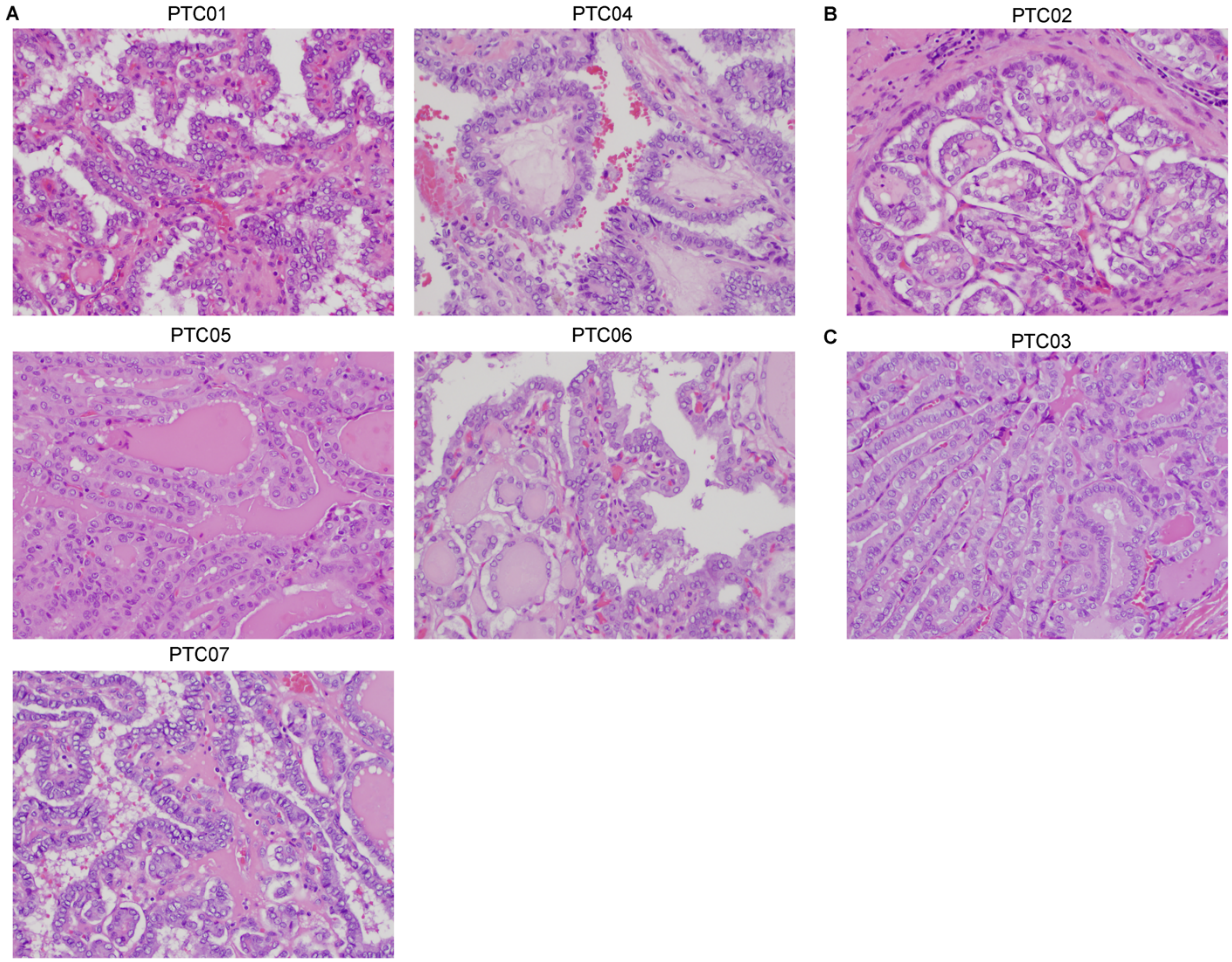

Supplemental Figure 1. Microscopic morphologies of 7 PTC tumors.
(A-C) Hematoxylin and Eosin (H&E) staining of 5 conventional (**A**), 1 follicular (**B**) and 1 tall cell (**C**) subtype for observations of microscopic morphologies.

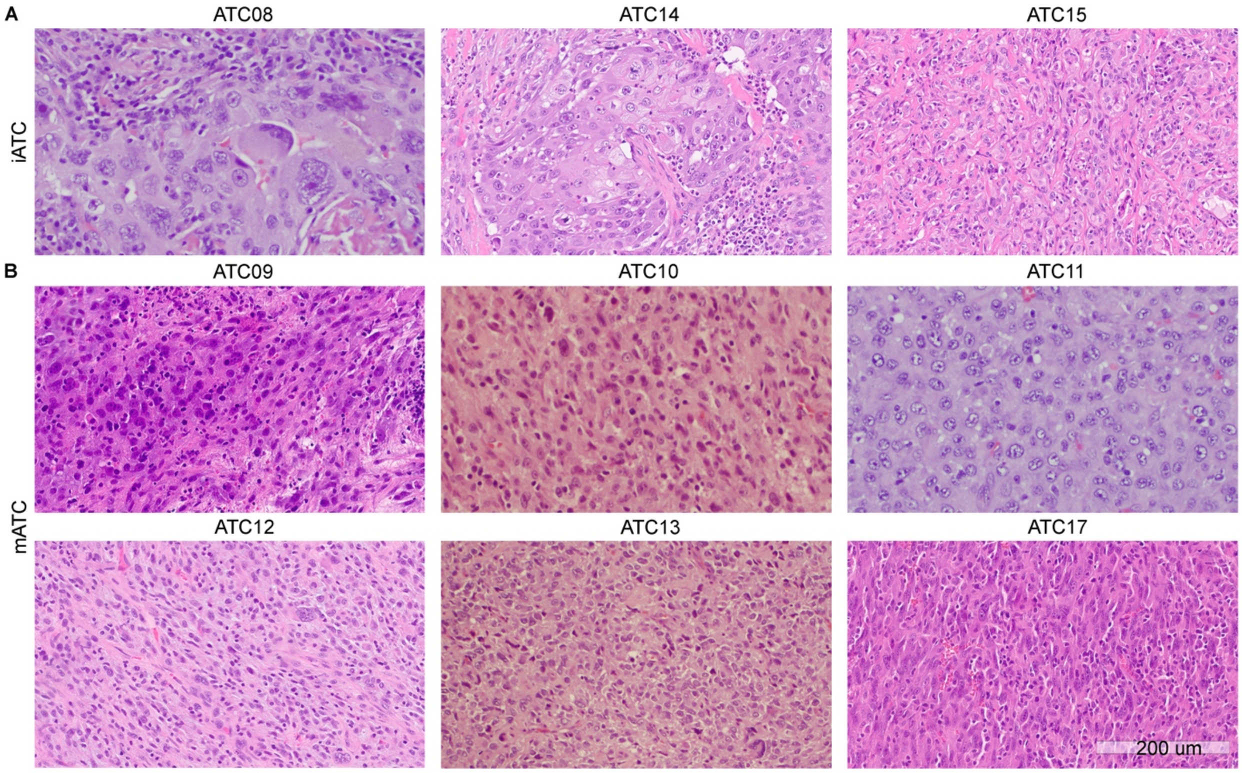

Supplemental Figure 2. Morphologies of 9 ATC tumors.
(A-B) Hematoxylin and Eosin (H&E) staining of 3 iATC tumors **(A)** and 6 mATC tumors **(B)** for observations of microscopic morphologies. Histological characteristics of all three iATC showed nested epithelial clusters with squamoid features with intervening stroma and mixed inflammation. The mATC samples in contrast show sheet-like growth of tumor cells often with spindled (mesenchymal) morphology.

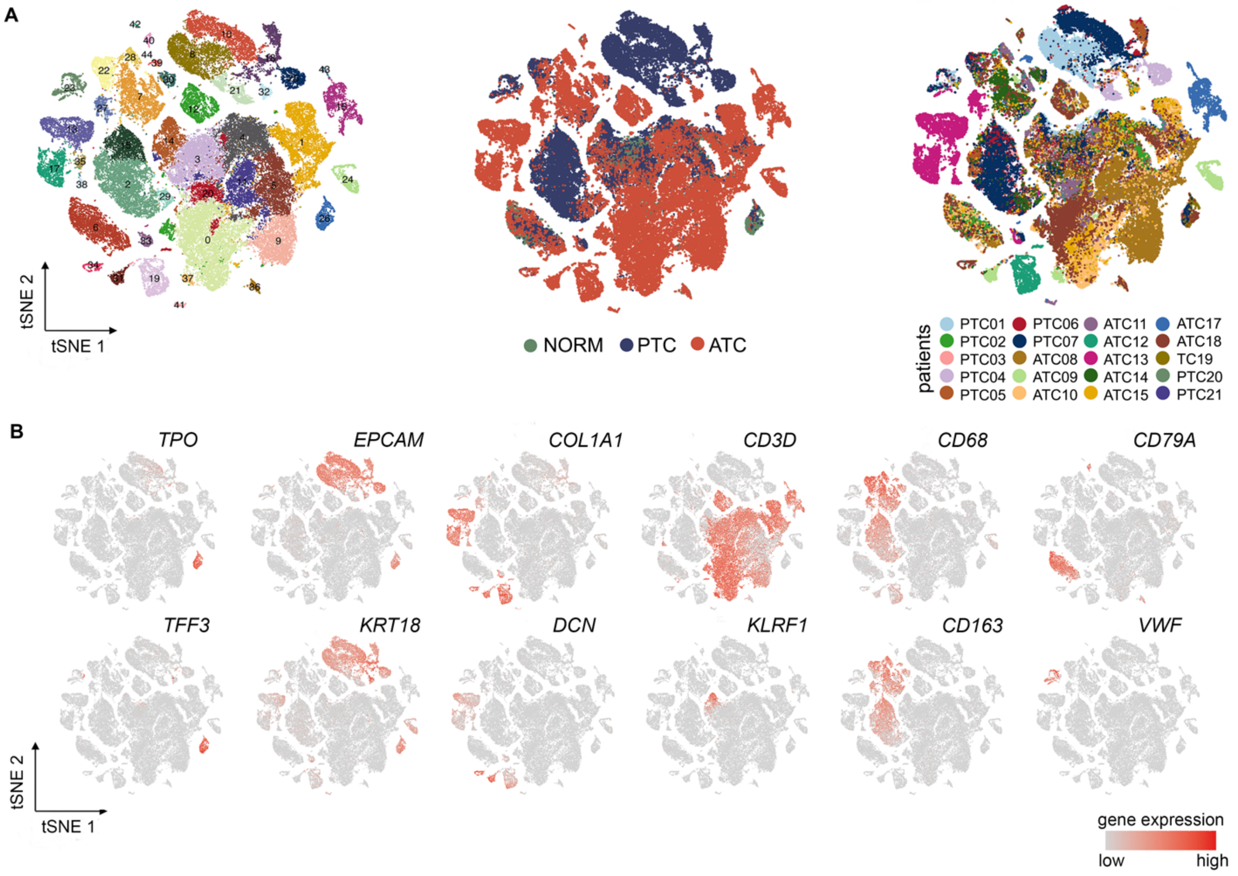

Supplemental Figure 3. scRNA-seq profiling of normal thyroids and tumor samples.
(A) t-SNE projection of single cells annotated with cluster IDs (left), tissue origins (middle) and patient IDs (right) of all samples. **(B)** tSNE showing expression levels of marker genes of eight major cell types.

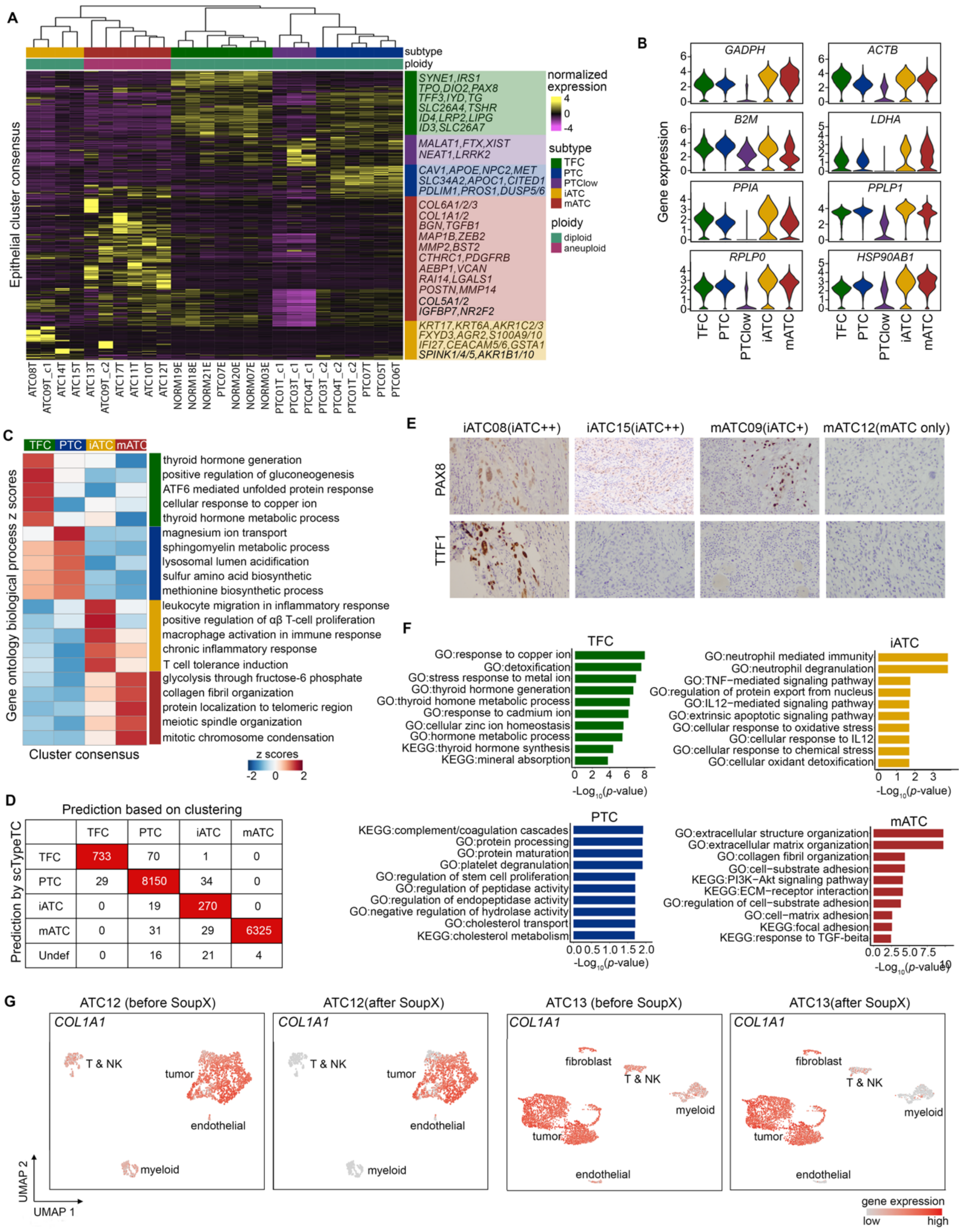

Supplemental Figure 4. Heterogeneous cancer cell subtypes in thyroid cancer.

(A) Heatmap showing unsupervised clustering of epithelial cluster consensus based on a union of DEGs from all patients. Top DEGs are indicated on the right. **(B)** Violin plots of normalized expression levels of housekeeping genes in 5 clusters of epithelial cluster consensus. **(C)** Heatmap of gene ontology (BP: biological process) z-scores of top differentially enriched gene sets based on GSVA analysis. **(D)** 2x2 table showing concordance between clustering and scTypeTC results. **(E)** IHC staining of thyroid differentiation proteins in 4 examples of ATC tumors. iATC++ indicates higher fractions of iATC cells, +, some iATC cells, a few iATC cell, -, no iATC cells. **(F)** Bar plots depicting the enriched BP terms and KEGG pathways of top 50 upregulated genes in 4 epithelial cell subtypes. **(G)** Comparisons of collagen gene expression levels before and after removal of ambient RNAs with 'SoupX'.

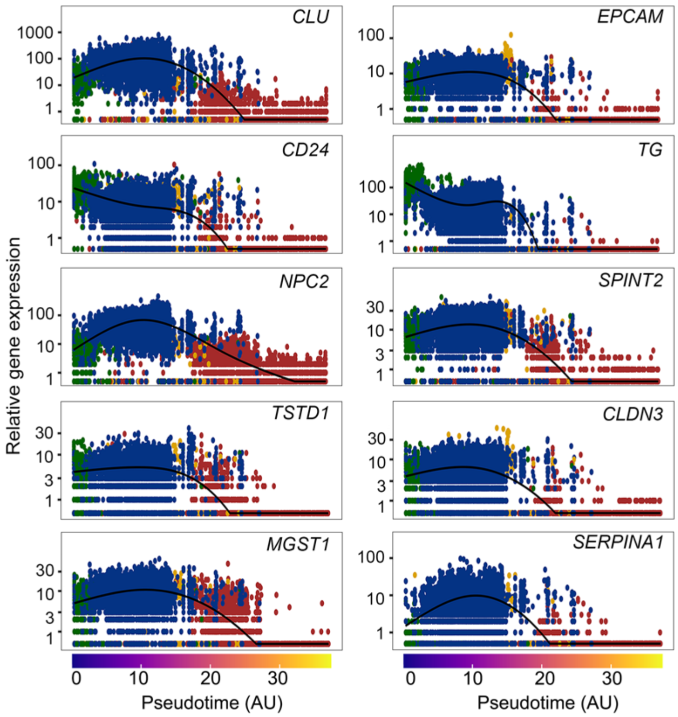

Supplemental Figure 5. Gene transcriptional programs underlying anaplastic transformation.
Scatter plots of top down-regulated genes that were significantly changed during ATC progression over pseudo-time. AU: artificial unit of pseudo-time.

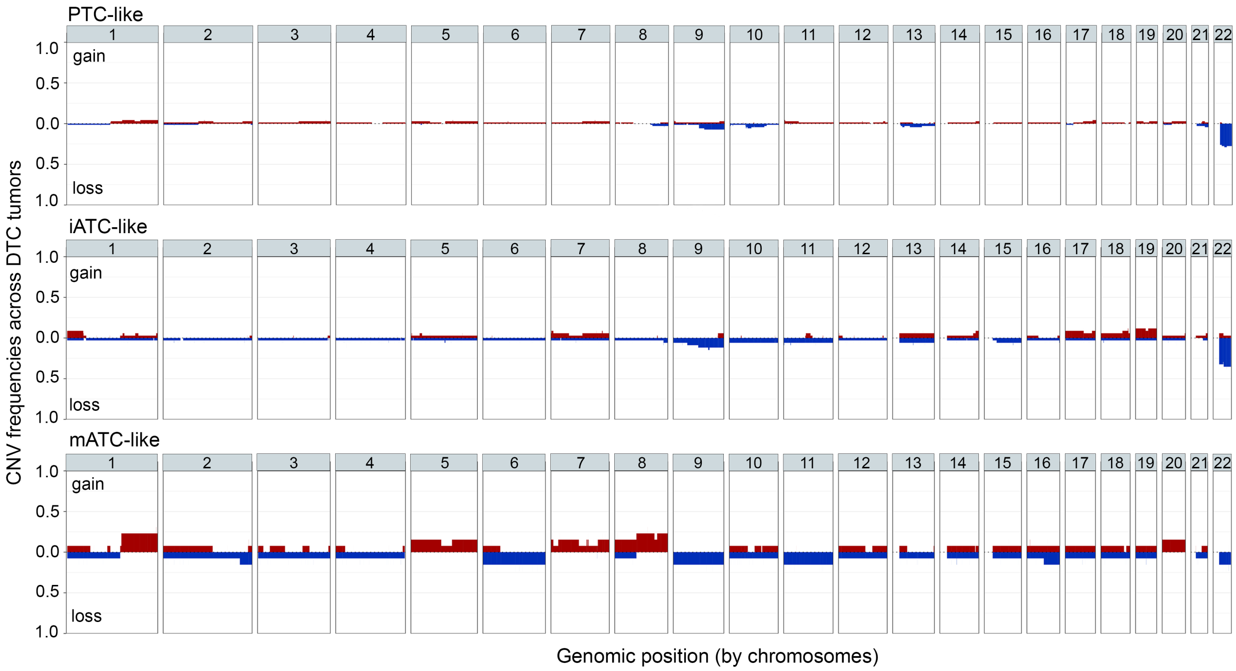

Supplemental Figure 6. DNA copy number alterations of DTC samples in the TCGA dataset.
Frequency plot of CNAs across PTC-like patients (top), iATC-like patients (middle) and mATC-like patients (bottom).

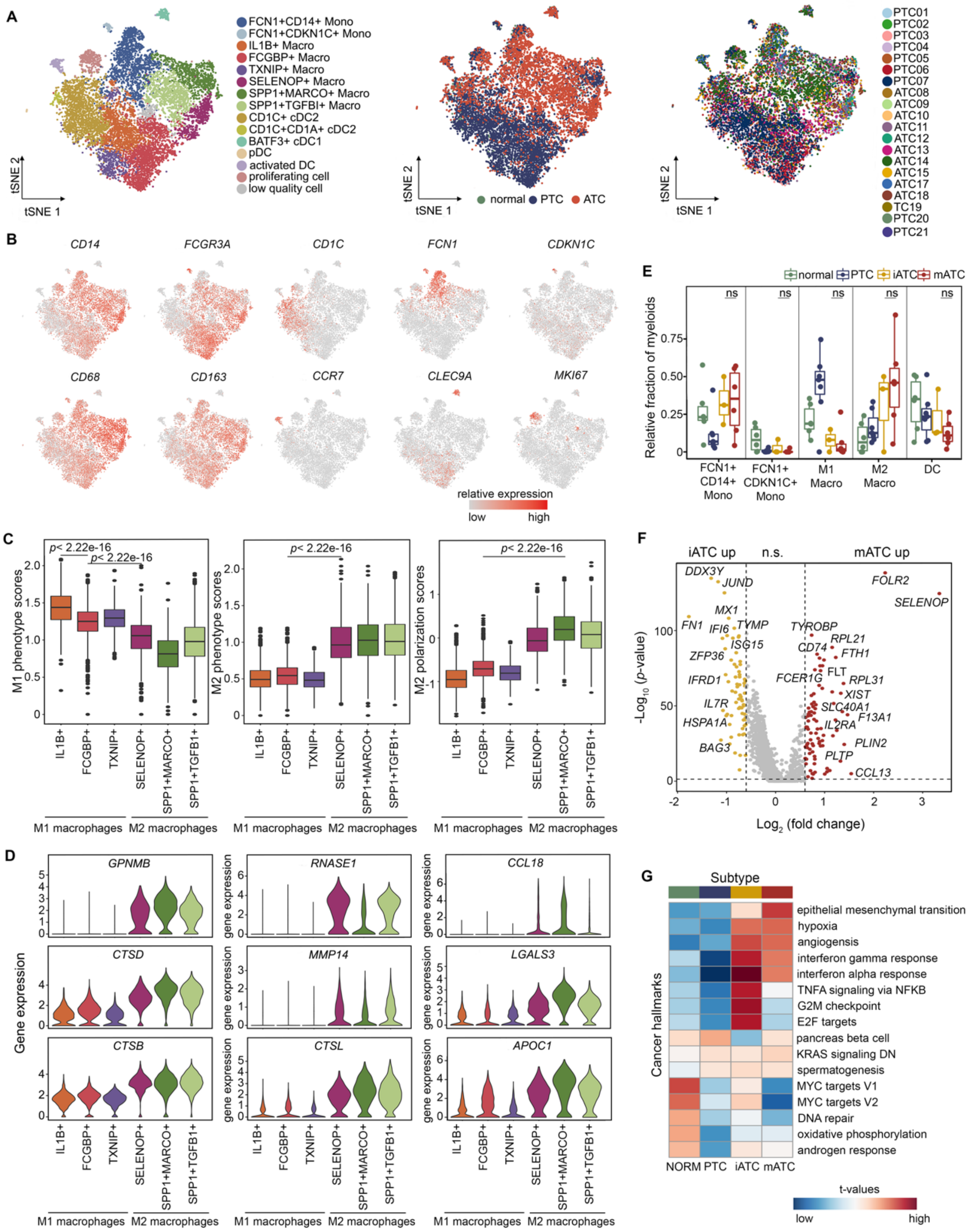

Supplemental Figure 7. Heterogeneous myeloid cells in normal thyroids and tumor tissues.
(A) t-SNE projection of subclustered myeloid cells derived from normal thyroid, PTC and ATC tissues, color-coded by subpopulations (left), tissue types (middle) and patient IDs (right). **(B)** t-SNE plots showing normalized expressions of selected marker genes in myeloid cell subtypes. **(C)** Boxplots showing comparison of M1 phenotype (left), M2 phenotype (middle) scores and M1 to M2 polarization (right) scores across 6 macrophage subtypes. *P-values*: WilCox test. **(D)** Violin plots of normalized expressions of newly derived M2 gene signature. **(E)** Boxplot of fractions of 5 major myeloid subtypes in 4 tumor subtypes. *P-values*: two-sided unpaired t test. **(F)** Volcano plot of differentially expressed genes between iATC and mATC-derived macrophages after 'SoupX'. *P-values*: WilCox test. **(G)** Top differentially enriched hallmark gene sets in macrophages of 4 subtypes by GSVA analysis with data processed by 'SoupX'. *ns*: not significant.

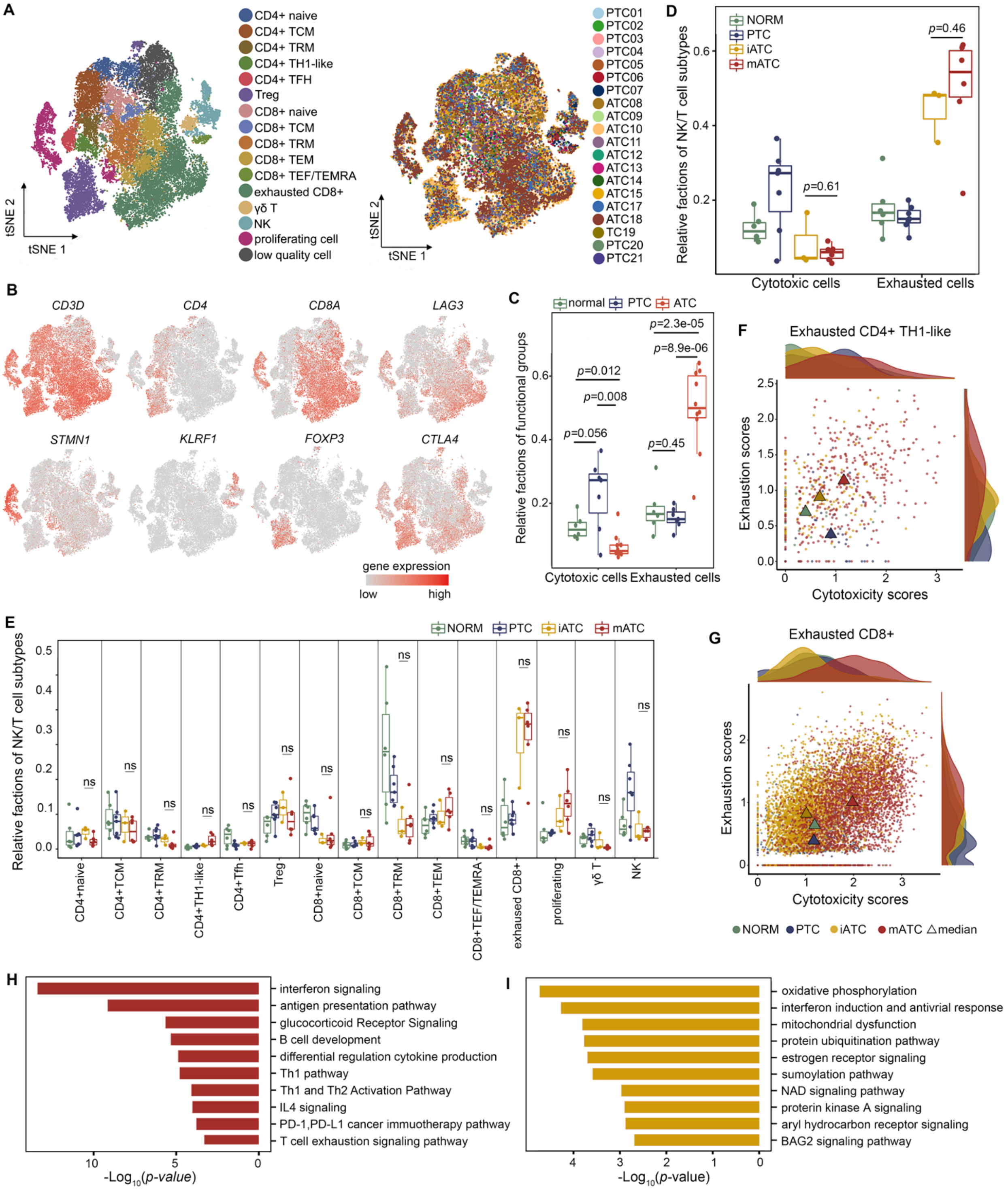

Supplemental Figure 8. T and NK cell subtypes in normal thyroids and tumor samples.
(A) t-SNE plot showing T and NK cells of normal thyroid, PTC and ATC samples, color-coded by subpopulations (left) and patient IDs (right). **(B)** t-SNE plots of normalized expressions of top marker genes of T and NK cell subtypes. **(C-D)** Boxplot of relative fractions of two major subgroups, cytotoxic and exhausted cells in normal thyroids, PTC and ATC samples **(C)** and in normal, PTC, iATC and mATC groups **(D)**. **(E)** Boxplots of relative fractions of all T and NK subtypes in normal thyroid, PTC, iATC and mATC samples. **(F-G)** Scatterplots of exhausted CD4+TH1-like cells (**F**) and exhausted CD8+T cells (**G**) phenotypes. Top and right subpanels show the density plots of phenotype scores**.** **(H-I)** Bar plots of enriched canonical pathways in exhausted CD8+T cells in iATC (**H**) and mATC (**I**). *P-values*: two-sided unpaired t test. ***, *P-value* < 0.001; **, *P-value* < 0.01; *, *P-value* < 0.05. *ns*: not significant.

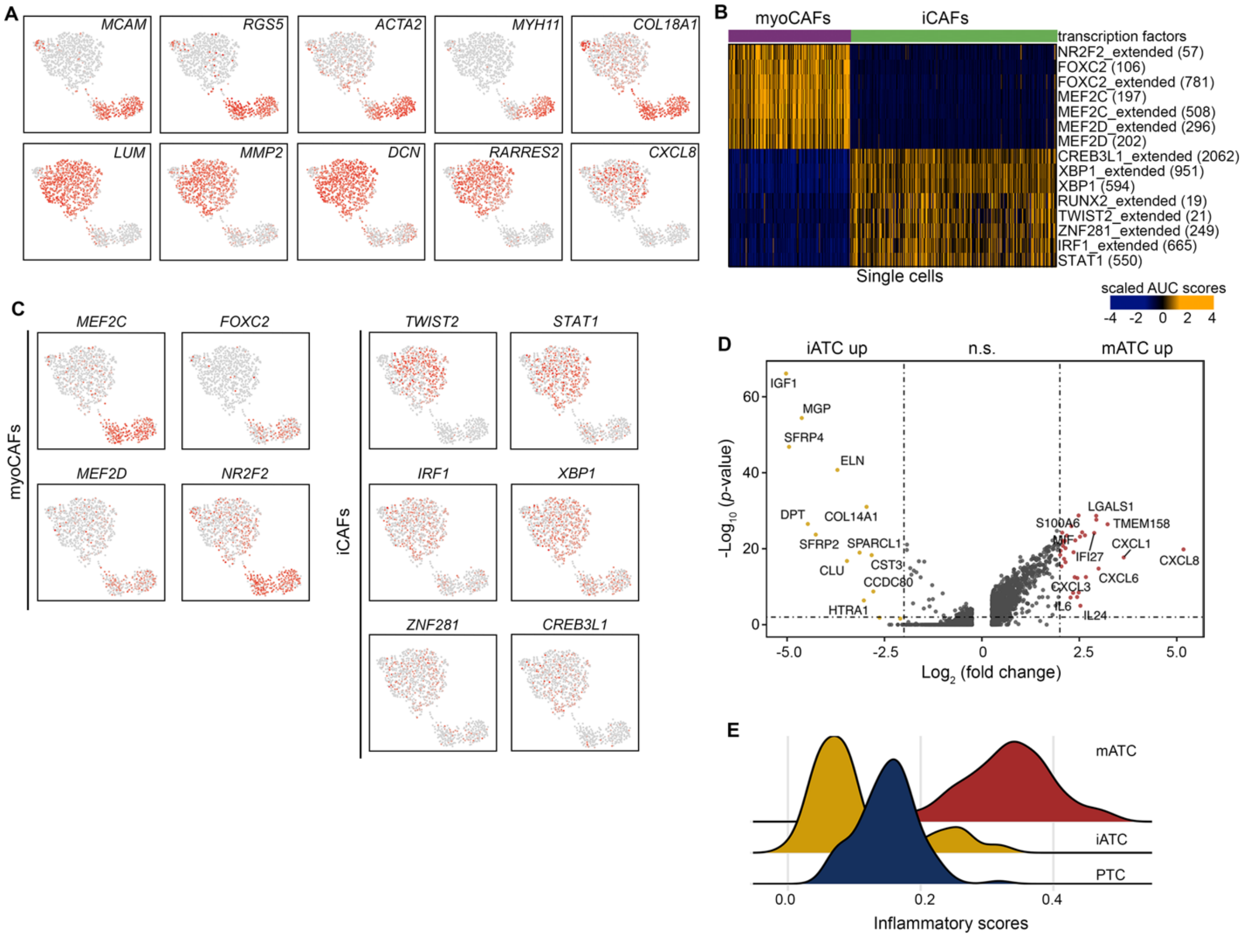

Supplemental Figure 9. Transcriptional regulations of CAFs in thyroid cancer patients.
(A) UMAP plots of selected marker gene expression z-scores of myoCAFs (top) and iCAFs (bottom). **(B)** Heatmap of area under the curve (AUC) of gene expression regulation by transcription factors estimated by 'SCENIC'. **(C)** UMAP plots showing normalized expression of top transcriptional factors detected by 'SCENIC'. **(D)** Volcano plot of differentially expressed genes of iCAFs in iATC and mATC samples. **(E)** Ridgeline plots of average expressions of inflammatory signature of iCAFs in PTC, iATC and mATC samples. *P-values*: WilCox test. *ns*: not significant.

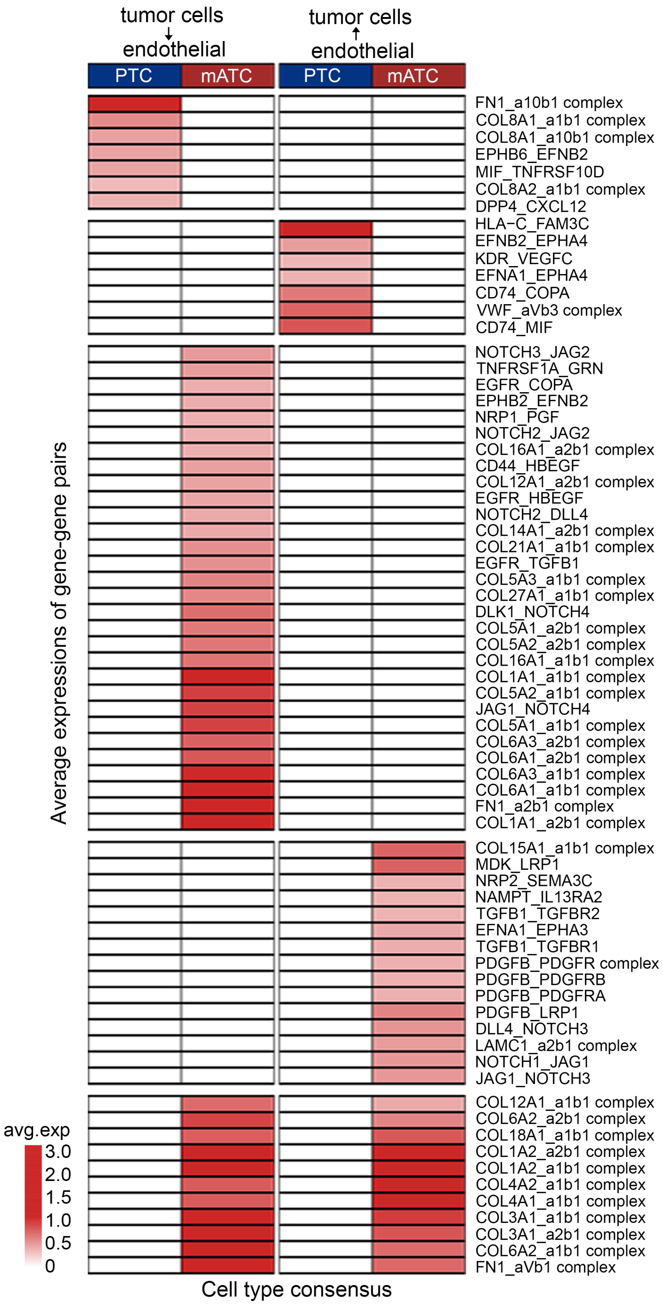

Supplemental Figure 10. Tumor cells-endothelial cells interaction network in thyroid cancer.
Heatmaps of average expressions of uniquely significant ligand-receptor pairs between tumor cells and endothelial cells. Significant interactions had *P-valuse* < 0.05, one sided permutation test, mean expression ≥ 0.5.

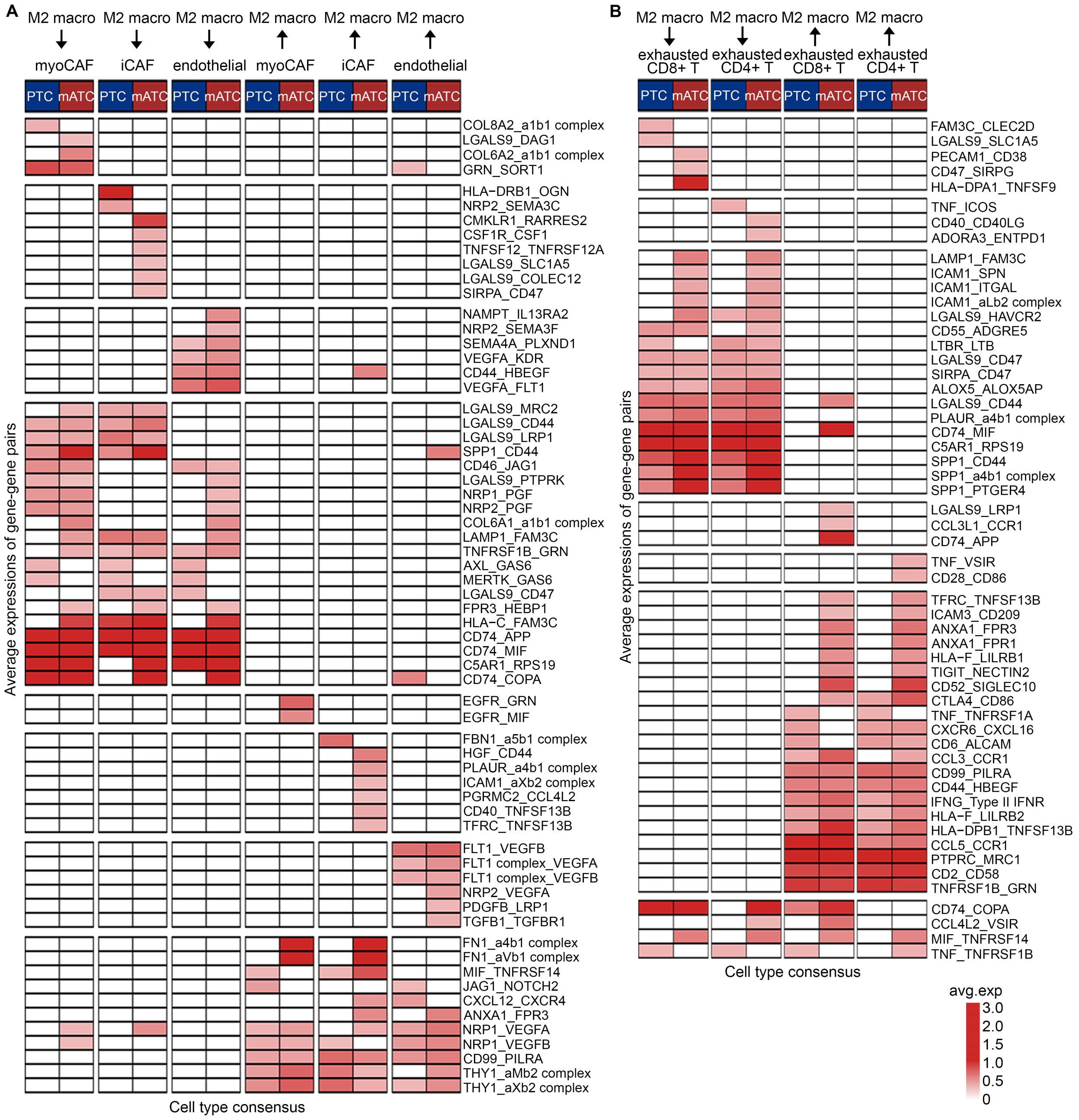

Supplemental Figure 11. Cell-cell interaction network associated with M2 macrophages in thyroid cancer. (A-B) Heatmaps of average expressions of uniquely significant ligand-receptor pairs between M2 macrophages and CAFs, M2 macrophages and endothelial cells (**A**), M2 macrophages and exhausted C8+ T cells, M2 macrophages and exhausted C4+ T cells **(B)** in PTC and mATC samples. The blank represents a nonsignificant interaction. Significant interactions had *P-valuse* < 0.05, one sided permutation test, mean expression ≥ 0.5.
